# From Biological Waste to Therapeutic Resources: A Comprehensive Review of Stem Cell Sources, Characterization, and Biomedical Potentials

**DOI:** 10.1007/s12015-025-10989-3

**Published:** 2025-10-15

**Authors:** Beatrice Camia, Manuela Monti

**Affiliations:** 1 Histology and Embryology Unit, Department of Public Health, Experimental and Forensic Medicine, University of Pavia, viale Forlanini 10, 27100 Pavia, Italy; 2 Research Center of Regenerative Medicine, IRCCS San Matteo Foundation for Hospitalization and Healthcare, Viale Golgi 19, Pavia, Italy

**Keywords:** Biological waste, Mesenchymal stem cell, Multilineage differentiation, Regenerative medicine, Extracellular vesicles, ATMP regulation, GMP manufacturing

## Abstract

The concept of utilizing biological waste as a resource dates back centuries, with early practices in traditional medicine repurposing discarded tissues for healing purposes. In recent decades, advances in stem cell biology have revitalized this concept by identifying multipotent stem cells within various waste materials, including urine, adipose tissue, follicular fluid, umbilical cord blood, fetal annexes, menstrual blood, and dental pulp byproducts. These sources offer a minimally invasive, ethically sound, and cost-effective alternative to conventional stem cell harvesting methods. Stem cells derived from waste materials exhibit robust proliferative abilities and multilineage differentiation potential, positioning them as valuable tools for regenerative medicine, tissue engineering, and personalized therapeutic applications. Clinical studies highlight their promise. For example, mesenchymal stem cells from adipose tissue and umbilical cord blood have shown safety and some effectiveness in early trials. These studies report improvements of up to 30–40% in recovery scores for osteoarthritis and ischemic heart disease, as well as a 20–35% decrease in inflammatory markers for autoimmune disorders. Cord blood stem cell transplants have shown 70–90% survival rates in children with blood cancers. This underscores the clinical potential of waste-derived stem cells. However, regulatory issues limit broader use. Agencies like the U.S. Food and Drug Administration and the European Medicines Agency classify many processing methods, especially enzymatic digestion, as “more-than-minimal manipulation.” This triggers strict requirements for Good Manufacturing Practice, clinical validation, and safety checks. These rules protect donors, ensure consistency, and check long-term safety. However, they also slow down clinical adoption. This review describes the history and recent advances in recycling biological waste to obtain stem cells, operating within the theoretical framework that positions waste-derived materials as viable sources for regenerative medicine. It highlights how these developments are transforming biomedical research and clinical care.

**Supplementary Information:**

The online version contains supplementary material available at 10.1007/s12015-025-10989-3.

## Introduction

### Historical view

*"Nothing is created, nothing is destroyed, everything is transformed"*

*Antoine Lavoisier*

The reuse of biological material—particularly substances once labeled as “waste”—represents a longstanding concept. Throughout history, biology has transformed materials once seen as useless or taboo into a valuable resource for medicine and research.

In ancient Greece and Rome, physicians and scholars such as Hippocrates, Galen, and Pliny the Elder, to cite a few [1, 2], viewed bodily fluids like blood, bile, and urine as diagnostic windows into health. They even incorporated substances such as earwax, menstrual blood, or discarded tissues into remedies [3]. Although these practices lacked a cellular understanding, they foreshadowed the therapeutic reuse of human material. By the middle Ages and Renaissance, anatomists made cadavers central to study. Pioneers such as Mondino de Liuzzi, Leonardo da Vinci, and Vesalius dissected bodies to advance knowledge of the human body [4, 5]. Later, in the 17th to 19th centuries, scientists studied discarded tissues from autopsies and surgeries under the microscope. This work laid the foundations of modern histology and pathology [6].

**Fig. 1:**
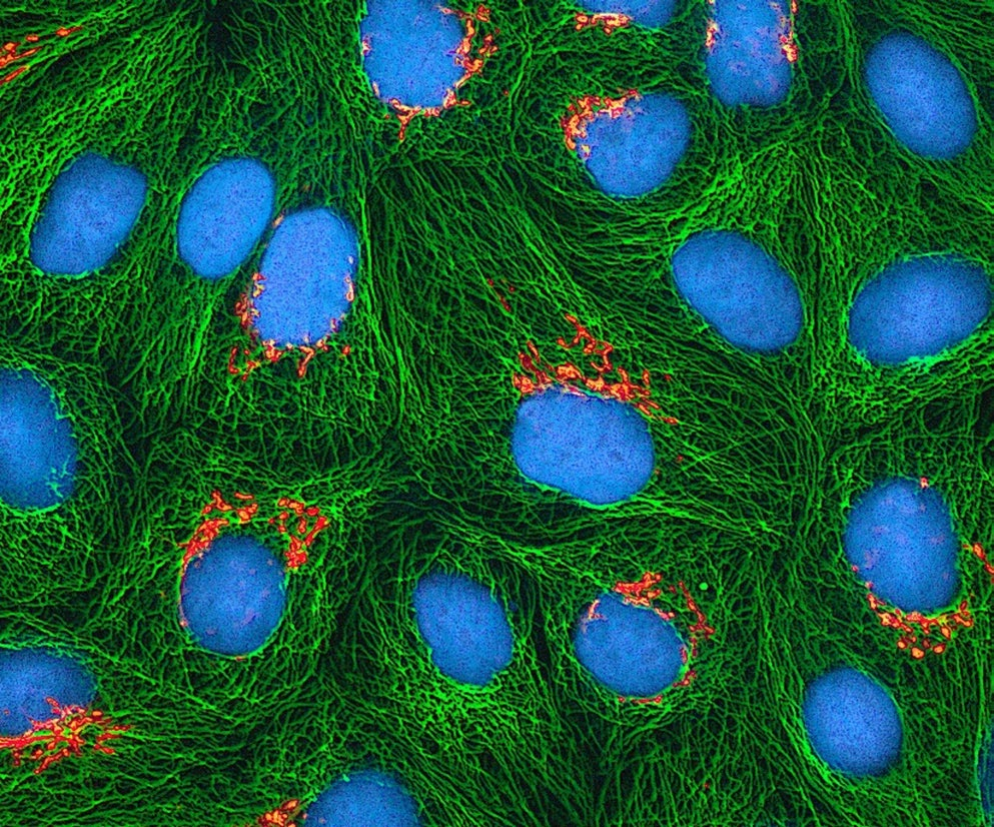
Multiphoton fluorescence image of cultured HeLa cells with a fluorescent protein targeted to the Golgi apparatus (orange), microtubules (green) and counterstained for DNA (cyan). Credit: Tom Deerinck, NIGMS, NIH. The Web site of the National Cancer Institute (https://www.cancer.gov)

The discovery of the HeLa cell line in 1951, the first immortal human cell line, had a profound impact on the 20th century (Fig. 1). It also marked the beginning of a new era in biomedical research and highlighted the ethical issues surrounding informed consent [7]. Since then, researchers have identified materials once discarded—such as placenta, umbilical cord blood, amniotic fluid, and foreskin—as abundant sources of stem cells with therapeutic potential.

Progress in stem cell biology, regenerative medicine, and biobanks establishment have confirms the biomedical value of these resources, and enables systematically collection and use for research, diagnostics, and therapies. Against this backdrop, this narrative review examines stem cell sources obtainable from biological waste, focusing on their characteristics, differentiation capacity, and clinical applications, and encourages further exploration of “hidden reservoirs” in discarded human materials. To structure this exploration, it is essential to outline the theoretical framework that underpins the review and the key questions that guide the analysis.

### Theoretical Framework and Guiding Questions

This review adopts a regenerative medicine framework that conceptualizes biological waste as a reservoir of stem cells with biomedical utility. The theoretical foundation is in stem cell biology, with a focus on self-renewal, multipotency, and paracrine signaling. It pairs these with translational perspectives that emphasize minimally invasive, ethically acceptable, and cost-effective alternatives to traditional stem cell sources. The review addresses five main questions: (1) which categories of biological waste contain stem cell populations of biomedical relevance? (2) What phenotypic markers, differentiation potential, and functional properties characterize these cells? (3) What therapeutic and research applications can they support? (4) What limitations and challenges affect their collection, standardization, and clinical translation? (5) How can exploiting these sources advance personalized and sustainable cell-based therapies?

#### Rationale for Source Selection

This review highlights eight biological waste materials, selected according to three principal criteria. First, they are clinically and ethically acceptable tissues or fluids, collected non-invasively (e.g., urine, menstrual blood) or as byproducts of routine medical procedures (e.g., follicular fluid, umbilical cord, fetal annexes, dental pulp). Second, these sources contain stem cell populations with multipotent properties, as demonstrated by phenotypic characterization experimental studies. Third, they show established or emerging translational potential in regenerative medicine, disease modeling, and immunomodulation.

These criteria differentiate the selected materials from other waste sources that yield insufficient or poorly characterized cell populations, present greater ethical or technical challenges, or lack robust evidence for their suitability. Focusing on these eight categories allows emphasis on stem cell reservoirs that are scientifically validated and relevant for future therapeutic development.

#### Literature Search and Inclusion Criteria

Searches in PubMed, Scopus, and Web of Science identified the reviewed literature. The search strategy combined the terms “stem cells,” “biological waste,” “urine,” “adipose tissue,” “follicular fluid,” “umbilical cord blood,” “placenta,” “Wharton’s jelly,” “menstrual blood,” and “dental pulp”. The review focused on references published primarily from 2000 to 2025 and incorporated earlier landmark studies when relevant to historical or conceptual framing.

Inclusion criteria were: (i) studies characterizing stem or progenitor cells isolated from the eight selected biological waste sources; (ii) reports providing phenotypic, differentiation, or functional data; and (iii) preclinical or clinical studies demonstrating translational applications. Exclusion criteria were: (i) studies lacking primary experimental data (such as opinion pieces without evidence); (ii) sources outside the defined eight waste materials; and (iii) reports with insufficient methodological detail to support reproducibility.

This structured approach ensured that the review remained focused on scientifically validated, ethically acceptable, and translationally relevant waste-derived stem cell sources.

The following section examines the fundamental biological properties of stem cells, providing a foundation for evaluating those derived from biological waste materials.

## Stem Cells

Stem cells are a population of highly undifferentiated cells characterized by their remarkable abilities for self-renewal, proliferation, and differentiation into mono- or multilineage cells. Due to these unique characteristics, stem cells have become increasingly central to regenerative medicine and cutting-edge biotechnologies in recent years. Their applications extend far beyond simple cell replacement, encompassing innovative therapeutic strategies that aim to repair and regenerate damaged tissues, modulate immune responses, and restore organ function. Additionally, stem cells serve as powerful models for investigating the pathogenic mechanisms underlying complex diseases, such as degenerative and autoimmune disorders, and for advancing novel pharmacological interventions, thereby establishing them as a cornerstone of translational medicine [8, 9].

Stem cells are categorized by their potency. Embryonic stem cells (ESCs) are pluripotent, able to form all cell types, while induced pluripotent stem cells (iPSCs) are reprogrammed somatic cells with similar capabilities [10, 11]. Adult (somatic) stem cells, found in postnatal tissues, are multipotent and support tissue maintenance by renewing cells within their germ layer, but cannot generate all cell types [12]. They avoid many ethical concerns and require only minimally invasive methods for collection although traditional sources, such as bone marrow, involve invasive procedures [13].

These limitations have prompted the search for alternative, more accessible sources of adult stem cell. Some sources use non-invasive collection methods. In contrast, some clinical interventions for diagnostic or therapeutic purposes yield stem cells through invasive procedure. Both approaches demonstrate the potential to repurpose biological waste as valuable scientific resources. Promising sources of stem cells from such waste include urine, adipose tissue, ovarian follicular fluid, umbilical cord blood, amniotic fluid, placenta, Wharton’s jelly, menstrual blood, and dental pulp (Table 1) [14–18]. These materials can be collected without harming donors and offer ethical, cost-effective options for regenerative therapies.

**Table 1:**
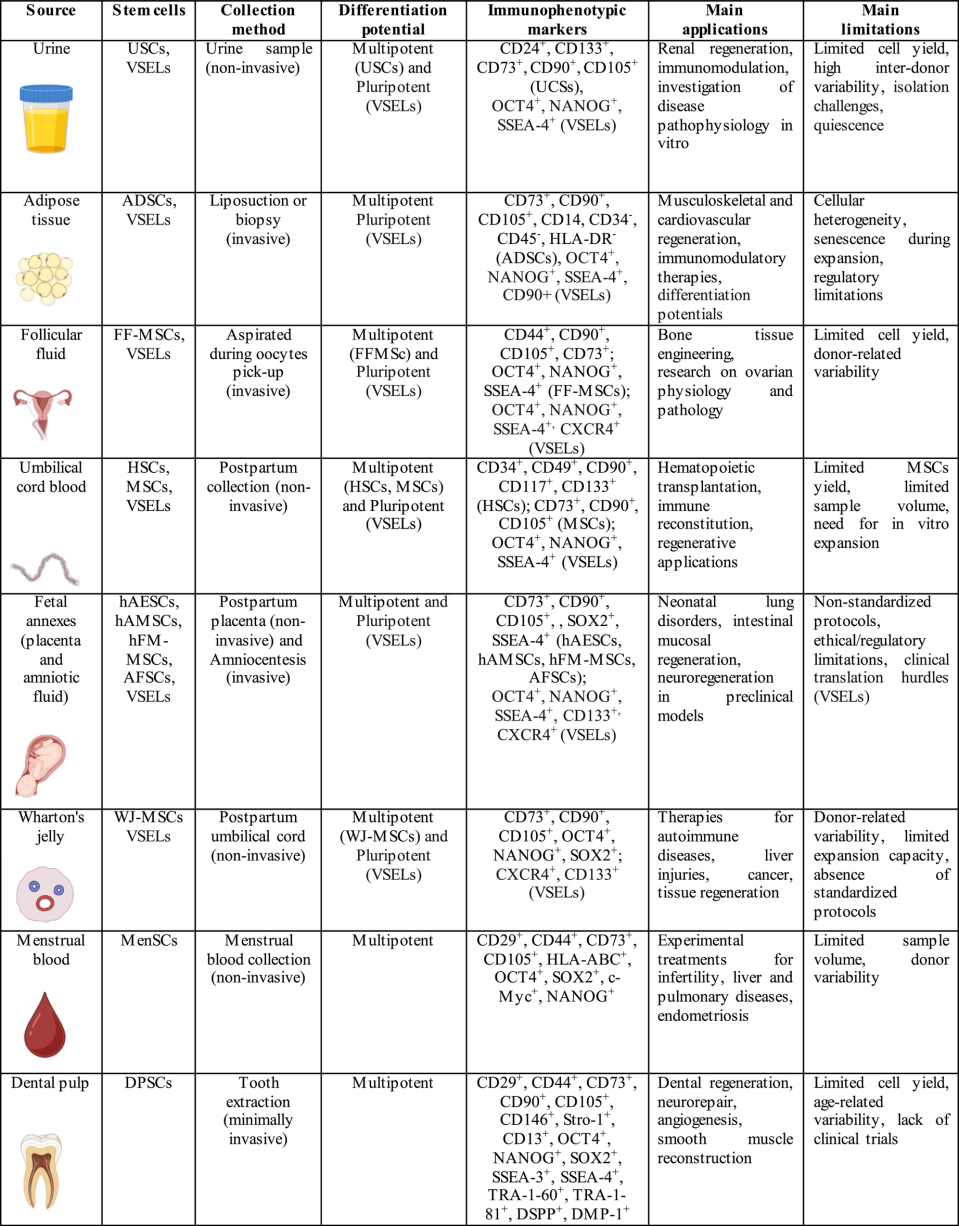
This table summarizes the principal characteristics of stem cells derived from waste biological materials, as presented in this review, in accordance with the rationale for source selection and the pre-specified inclusion criteria

Abbreviations: USCs: urine-derived stem cells; ADSCs: adipose-derived stem cells; FF-MSCs: follicular fluid-derived mesenchymal stem cells; HSCs: hematopoietic stem cells; MSCs: mesenchymal stem cells; VSELs: very small embryonic-like stem cells; hAESCs: human amniotic epithelial stem cells; hAMSCs: human amniotic mesenchymal stem cells, hFM-MSCs: mesenchymal stromal cells from the amniochorionic membrane; AFSCs: amniotic fluid stem cells; WJ-MSCs: Wharton’s jelly mesenchymal stem cells; MenSCs: menstrual blood-derived stem cells; DPSCs: dental pulp stem cells. Icons are created in BioRender.com

### Biological Waste Materials

#### Urine

Urine is traditionally considered a biological waste fluid—the final product of renal filtration—with the primary physiological role of eliminating metabolic waste substances such as urea, creatinine, and uric acid from the body. Additionally, urine helps maintain fluid-electrolyte balance and overall homeostasis. Recent research, however, has prompted a significant reevaluation of urine’s functions. Urine no longer carries the sole label of metabolic waste; instead, its biologically active properties interact with urinary tract tissues, particularly the urothelium. Furthermore, urine carries functional molecules and viable cells with therapeutic potential [19]. This evolving understanding opens new avenues for exploring urine-derived cells and bioactive components in regenerative medicine and disease diagnostics.

Urine is composed primarily of water, accounting for approximately 95% of its volume, while the remaining 5% consists of dissolved solutes. Beyond these basic components, urine also contains proteins, extracellular microvesicles - including exosomes- and exfoliated epithelial cells from the urinary tract. Notably, among there exfoliated populations is a specialized subgroup of cells known as urine-derived stem cells (USCs). Recent studies have highlighted the therapeutic and diagnostic potential of USCs, thereby positioning urine not merely as waste but as a valuable biological resource for precision medicine and regenerative applications [20, 21].

Zhang and collaborators first identified USCs in 2008, demonstrating their potential as a source for urological tissue reconstruction [22]. Although their precise origin remains incompletely understood, USCs are believed to originate primarily from parietal epithelial cells of the glomerulus and exhibit varying degrees of stemness and multipotency once isolated [21].

USCs are multipotent adult stem cells that can be easily harvested from the urinary sediment of midstream samples via a completely non-invasive, cost-effective (less than $70 per sample) [23], painless, and repeatable procedure.

During initial isolation, even small urine volumes provide an average of 2–7.2 cells per 100 milliliters. These cells readily expand and form cellular clones.

These clones are capable of significant in vitro expansion, reaching up to 0.5 to 1 million cells within 10–15 days [22]. The culture media for USCs expansion typically included DMEM/F12 or Keratinocyte Serum-Free Medium, supplemented with Epidermal Growth Factor, Bovine Pituitary Extract, and Fetal Bovine Serum [24, 25].

These cells maintain genetic stability, exhibit a stem cell phenotype, and demonstrate active telomerase expression over time- features crucial for cellular longevity and therapeutic viability [21, 26].

Moreover, USCs are positive for a range of markers, including CD24, CD133, CD73, CD90, and CD105, and exhibit remarkable differentiation plasticity toward multiple cell lineages, such as osteogenic, myogenic, adipogenic, neuronal, and urothelial [27, 28].

This plasticity makes them valuable in tissue regeneration, not only through direct differentiation but also via indirect therapeutic mechanisms mediated by paracrine signaling. Indeed, USCs exhibit potent paracrine activity by secreting a variety of biologically active substances, including cytokines, growth factors, and exosomes with trophic and immunomodulatory properties [21, 27]. Among these, exosomes - small extracellular vesicles (EVs) ranging from 30 to150 nm- have garnered particular attention due to their heterogeneous and bioactive molecular cargo. These vesicles modulate immune responses, reduce oxidative stress, promote angiogenesis, regulate apoptosis, and enhance tissue repair in pathological conditions such as ischemia and inflammation [29].

Additionally, USC-derived exosomes display strong antioxidant activity and exert protective effects in pathological models such as diabetic nephropathy and chronic kidney disease [28].

USCs also play critical roles in modulating immune responses and apoptosis. Notably, they can inhibit CD4 + T-cell proliferation (useful in treating inflammatory and autoimmune diseases) and help regulate the balance between pro-apoptotic and anti-apoptotic proteins [28].

Compared to other stem cell sources such as ESCs or iPSCs, USCs demonstrate a superior safety profile, exhibiting a very low risk of tumorigenicity [21, 30]. Kim and collaborators [30] conducted a study to investigate the safety and bio-distribution of Klotho (kidney-specific therapeutic factor)-enhanced human USCs in BALB/c nude mice. Their goal was to advance these cells for clinical applications in kidney diseases. After both single and repeated intravenous administration of USCs, the researchers observed that Klotho-enhanced USCs targeted injured renal tissue and restored Klotho expression in models of ischemia–reperfusion injury. This demonstrated significant therapeutic potential. Importantly, there were no signs of toxicological or immunological adverse effects in the treated animals. Long-term monitoring over twenty-six weeks found no evidence of tumorigenicity attributable to the transplanted USCs. The few tumors observed were of murine rather than human origin, consistent with spontaneous tumor formation common in this mouse strain.

From a diagnostic perspective, USCs can be used to generate patient-specific cellular models for studying renal diseases and identifying urinary biomarkers associated with chronic or degenerative kidney disorders [21, 27]. Their non-invasive collection and strong biological relevance make them suitable for predictive diagnostics and in vitro drug toxicity screening [15, 31].

The ability to isolate USCs from the same individual at different time points enables longitudinal studies and personalized medicine applications, including patient-specific disease modeling and pharmacological screening [27]. Despite these advantages, the clinical use of USCs still faces some limitations. Cell yield can vary significantly among individuals influenced by factors such as age, health status, diet, and hydration. In some cases, urine samples may contain insufficient numbers of viable cells, necessitating the collection of large or repeated volumes. Additionally, standardization of isolation and culture protocols remains an ongoing concern, as USCs may exhibit phenotypic heterogeneity based on collection and culture conditions [28].

Lastly, although they present a low tumorigenic risk, long-term safety still requires comprehensive evaluation.

In conclusion, urine is not only an easily accessible and non-invasive biological fluid but also a safe and versatile source of adult stem cells. UCSs hold great promise across regenerative, predictive, and diagnostic medicine, though further research is needed to optimize their clinical application.

#### Adipose Tissue

Adipose tissue, often discarded as surgical waste, represents a promising and accessible source of adult stem cells. Liposuction and biopsy are two methods that supply this kind of tissue. The former, while more invasive, enables the collection of larger tissue volumes, making it suitable for applications that require high cell yields. Notably, different Body Mass Index (BMI) values significantly influence the isolation of adipose-derived stem cells (ADSCs). Specifically, ADSCs derived from individuals with higher BMIs exhibit slower proliferation rates, increased inflammatory profiles, altered gene expression related to aging, and higher levels of certain inflammatory markers, such as CCL2 (C-C-motif Chemokine 2) [32]. Although the adipogenic potential of these cells can vary, suggesting potential but compromised differentiation, the overall functional differences in ADSCs from various BMI groups highlight their altered biological properties and potential impact on metabolic health and regenerative medicine applications [32].

In contrast, biopsy is a less invasive method that allows for the targeted collection of small tissue samples. This approach offers particular advantages in clinical scenarios that require minimal tissue harvesting.

Adipose tissue comprises a highly heterogeneous population of cells, including preadipocytes, pericytes, hematopoietic cells, fibroblasts, smooth muscle cells, endothelial cells, and immune cells (such as B and T lymphocytes, macrophages, and myeloid cells), along with ADSCs [14]. ADSCs reside within the stromal vascular fraction of adipose tissue, a complex microenvironment primarily composed of connective tissue and blood vessels that supports the adipocytes.

Once collected, ADSCs can be isolated using various techniques, with enzymatic digestion typically using collagenase or Liberase, or by mechanical processing methods such as microfragmentation, centrifugation, or filtration, and while both approaches target the same stromal vascular compartment, the products they generate are not equivalent [33].

Enzymatic digestion reliably produces higher yields of viable nucleated cells, enriched in clonogenic mesenchymal progenitors, and offers a single-cell suspension suitable for expansion, precise dosing, and standardized phenotypic analysis. Optimization studies confirm that careful modulation of enzyme concentration and exposure enhances recovery while maintaining stemness properties [34, 35]. Mechanical isolation, in contrast, generates a lower number of liberated cells but preserves extracellular matrix scaffolds and perivascular niches, producing microfragmented adipose tissue (MFAT) that maintains a native structural context argued to support paracrine signaling and engraftment [36]. This distinction is more than technical, as enzymatically derived ADSCs suspensions and mechanically produced MFAT differ in formulation and thus in potential mechanisms of action. Regulatory frameworks also amplify these contrasts: in Europe and the United States, authorities generally classify enzymatic digestion of adipose tissue as more-than-minimal manipulation, which requires advanced manufacturing controls, whereas they often allow mechanical methods under minimal manipulation standards, making them more accessible for point-of-care autologous therapies [37]. As defined by the US Food and Drug Administration, more-than-minimal manipulation standards refer to human cells, tissues, and cellular or tissue-based products that have undergone processing that alters their original relevant characteristics, functions, or utility, while minimal manipulation standards mean processing that does not alter the relevant biological characteristics of cells.

The similarities indicate that each technique taps into the same cellular reservoir, yet their differences—in yield, product form, regulatory classification, and clinical effect profile— underscore that researchers should consider enzymatic and mechanical isolation of human adipose stem cells complementary rather than interchangeable strategies [34].

**Fig. 2:**
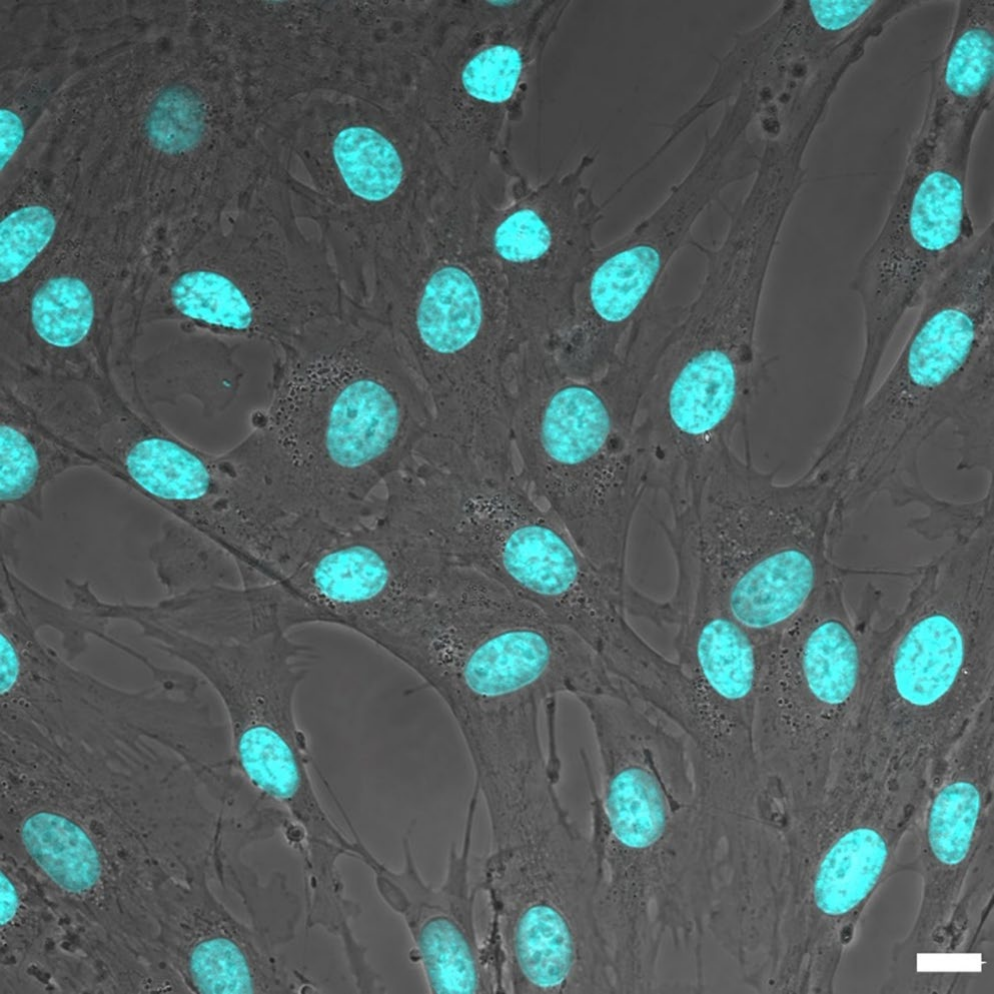
Phase contrast image of human ADSCs. Nuclei are stained in blue (DAPI). Magnification: 60X, bar: 10 μm. (Unpublished data)

ADSCs are mesenchymal stem cells (MSCs) with multipotent differentiation capacity and several biologically relevant characteristics. They can differentiate predominantly toward the mesodermal lineage into osteoblasts, chondrocytes, adipocytes, and myocytes, and they exhibit robust in vitro proliferation while maintaining genomic and phenotypic stability [38]. In culture, ADSCs adhere readily to plastic surfaces, assume a fibroblast-like morphology, and express an MSCs immunophenotype positive for CD73, CD90, and CD105, and negative for CD14, CD34, CD45, and HLA-DR (Fig. 2) [14, 33].

Beyond their differentiation potential, ADSCs exert therapeutic effects via paracrine mechanisms. They secrete a variety of cytokines and growth factors, including VEGF, HGF, TGF-β, IGF-1, as well as exosomes enriched with microRNAs and bioactive proteins. The secretions contribute to modulating immune responses, enhancing angiogenesis, regulating apoptosis, and promoting tissue regeneration [39] Among all biologically derived waste materials, adipose-derived stem cells (ADSCs) are among the most extensively studied in both preclinical and clinical settings. In regenerative medicine, they have shown efficacy in bone and cartilage repair, including treatment of bone defects, osteoarthritis, and joint injuries [38, 39]. Their angiogenic, anti-apoptotic, and anti-fibrotic effects are notably in cardiovascular and ischemic diseases such as myocardial infarction and peripheral ischemia. Their immunomodulatory capabilities support applications of ADSCs in models of autoimmune and inflammatory diseases, including multiple sclerosis, lupus, Crohn’s disease, and rheumatoid arthritis [14] Furthermore, ADSCs-derived exosomes have emerged as a promising cell-free therapeutic option. These exosomes, rich in miRNAs and bioactive factors, have demonstrated regenerative potential in wound healing, musculoskeletal repair, as well as in dermatological applications [40] Overall, ADSCs are a highly versatile therapeutic tool with broad, still partially untapped, potential. However, ADSCs also present certain limitations. Donor variability and cellular heterogeneity pose challenges to treatment standardization [41]. Additionally, pathological conditions such as obesity and aging can impair cell vitality and function. Extended in vitro expansion leads to senescence, diminishing their regenerative potential over time [42]. Moreover, concerns regarding safety and manipulation persist, and regulatory constraints continue to slow clinical translation [43].

#### Follicular Fluid

**Fig. 3:**
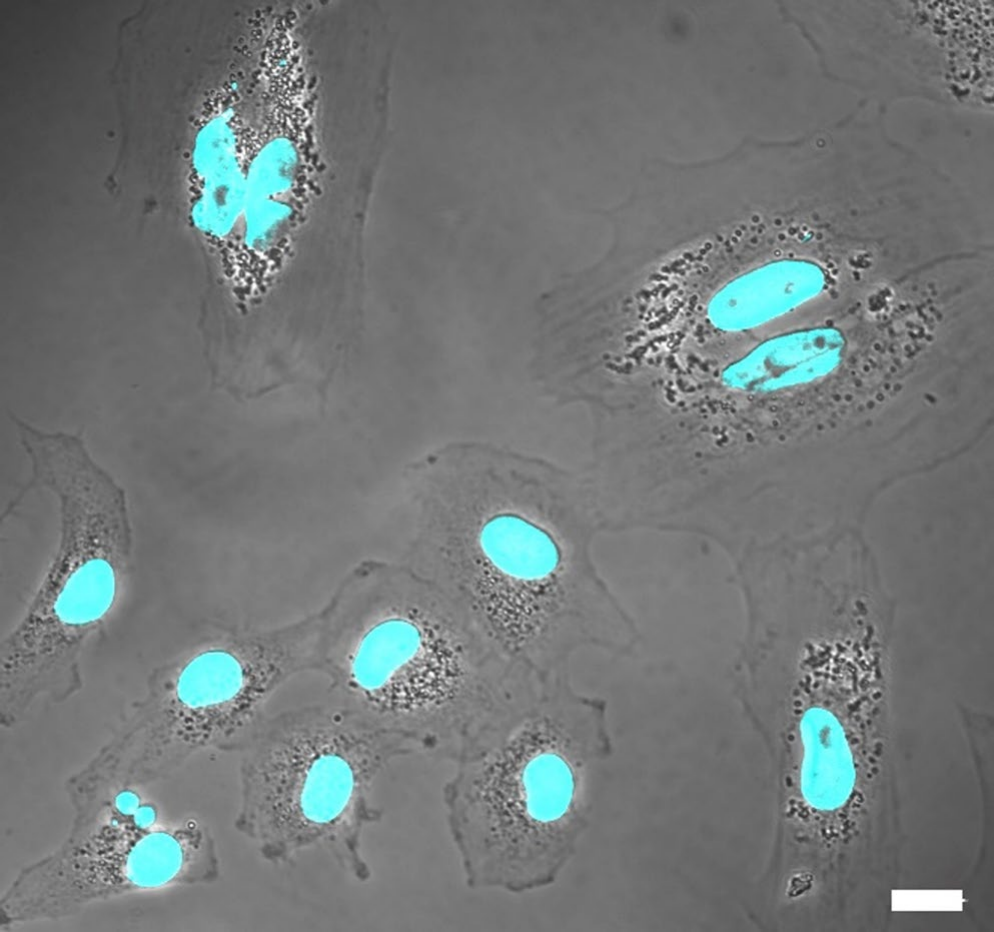
Phase contrast image of human FF-MSC. Nuclei are stained in blue (DAPI). Magnification: 60X, bar: 15 μm. (Unpublished data)

 Follicular fluid (FF) is the biological fluid found within mature ovarian follicles, the functional units of the ovary that support the development and maturation of the oocyte Granulosa cells and theca interna cells secrete the FF that contains a complex and dynamic mixture of bioactive components, including hormones, proteins and peptides, metabolites, lipids, growth factors and cytokines, antioxidants and reactive oxygen species (ROS), extracellular vesicles, and diverse cell populations [44–46]. Due to its intricate composition, FF plays a crucial role in establishing the biochemical environment required for oocyte maturation and in mediating communication between the oocyte and surrounding somatic cells During assisted reproduction technologies, particularly in vitro fertilization, FF is collected as part of the oocyte retrieval process following controlled ovarian stimulation. However, it is typically discarded- despite being a valuable source of cells and bioactive molecules Post-retrieval, the cellular component of the FF is notably heterogeneous. It includes granulosa cells, immune cells, and a population of MSC-like stem cells, as well as a minor fraction of epithelial cells likely introduced from the vaginal, cervical, or tubal epithelium during aspiration [47] Upon isolation, MSCs derived from follicular fluid (FF-MSCs) adhere to culture substrates such as plastic and glass, exhibiting progressive morphological changes. Initially rounded in shape, these cells gradually become cuboidal during early adhesion phases and eventually adopt an elongated, fibroblast-like morphology typical of MSCs (Fig. 3) [47, 48].

 From an immunophenotypic perspective, FF-MSCs express surface characteristics of MSCs, including CD44, CD90, CD105, and CD73. In addition, a minority of the cell population may express pluripotency-associated markers such as OCT4, NANOG, and SSEA-4, although the functional implications of this expression remain under investigation [47] It is crucial to recognize that the composition and properties of follicular fluid are highly influenced by the physiological and pathological status of the ovary. Conditions such as polycystic ovary syndrome (PCOS), advanced maternal age, oxidative stress, and chronic inflammation can significantly alter the biochemical profile of FF, including concentrations of hormones, growth factors, cytokines, ROS, extracellular matrix proteins, and exosomes, which in turn affect the vitality and phenotypic traits of isolated FF-MSCs [49].

Metabolomics analyses have revealed that FF from patients with endometriosis, PCOS, or diminished ovarian reserve displays altered levels of key energetic metabolites and nutrients, potentially impairing oocyte quality and the functional properties of associated stem cells [49].

In particular, proteomic profiling of small antral follicles in polycystic ovaries has shown increased expression of inflammatory and immune-related pathways, along with reduced levels of proteins critical for oocyte maturation and endocrine signaling [50]. These alterations may negatively influence the differentiation potential and regenerative capabilities of FF-MSCs.

Despite these influences, FF-MSCs have demonstrated multipotent differentiation capacity in vitro. Under appropriate induction conditions, they can differentiate into osteogenic, adipogenic, and chondrogenic lineages. A small subpopulation has also been reported to acquire epithelial- or neural-like morphologies during culture, suggesting morphological plasticity. However, conclusive evidence for transdifferentiation into ectodermal or endodermal lineages remains limited [47, 48, 51].

Given their properties, FF-MSCs show considerable promise in regenerative medicine. Their paracrine activity relies on the secretion of pro-regenerative, angiogenic, and immunomodulatory factors. Researchers have used these properties in models of bone tissue regeneration [52, 53].

FF-MSCs also serve as a valuable in vitro model for reproductive and toxicological research because their phenotypic and molecular characteristics partly reflect the follicular microenvironment from which they originate. FF-MSCs are useful for studying ovarian pathophysiology, assessing environmental or pharmacological exposures, and evaluating therapeutic interventions in a physiologically relevant human model, offering an ethically preferable alternative to animal testing [47, 54].

An emerging and particularly promising area of study is the analysis of FF-MSCs- derived exosomes. These EVs carry a molecular cargo rich in proteins, lipids, mRNAs, and microRNAs, with potential roles in modulating inflammation, promoting angiogenesis, and supporting tissue regeneration [55]. Nevertheless, the clinical translation of FF-MSCs faces several obstacles. The yield of viable stem cells per FF sample is typically low, and donor-dependent variability further complicates the standardization of these cells. Moreover, there remains a lack of consensus on optimal protocols for their isolation, expansion, and characterization. Until these challenges are resolved, the use of FF-MSCs will remain largely confined to the preclinical and experimental stages.

#### Umbilical Cord Blood

Umbilical cord blood (UCB) is the residual blood remaining in the placenta and umbilical cord following childbirth. Traditionally considered a biological waste product, UCB is now recognized as a rich and ethically accessible source of both pluripotent and multipotent stem cells [17].

Its collection is safe, non-invasive, and painless for both mother and newborn. Immediately after delivery and cord clamping, the procedure involves inserting a sterile needle into the umbilical vein. This approach yields between 60 and 100 ml of blood, with the amount depending on various maternal and perinatal factors UCB is biologically rich, containing a diverse population of hematologic and immune cells. These include T and B-lymphocytes, monocytes, macrophages, natural killer (NK) cells, granulocytes, nucleated red blood cells, and platelets. Among its most valuable components are hematopoietic stem cells (HSCs) and hematopoietic progenitor cells (HPSc), which have potent proliferative and regenerative capabilities [56, 57].

In smaller quantities, UCB also harbors MSCs, endothelial and epithelial progenitors, and regulatory immune cell subsets [58–60].

HSCs and HPCs from UCB express specific surface markers such as CD34, CD49, CD90, CD117, and CD133. Of particular interest is the CD34^+^CD38^−^ subpopulation, which represents a highly primitive stem cell phenotype. This population is significantly more abundant in UCB than in adult bone marrow, suggesting that UCB-HSCs possess enhanced capacity and distinct biological features [61].

Functionally, UCB-derived HSCs demonstrate superior hematopoietic reconstitution in vivo and greater proliferative potential in vitro. These cells have longer telomeres, contributing to enhanced replicative lifespan, and a faster transition out of the quiescent G0/G1 phase, enabling more rapid proliferation than adult-derived HSCs [58].

Such features have positioned UCB as a preferred source of stem cells for clinical transplantation, especially in the context of allogeneic hematopoietic stem cell transplantation. To date, regulatory agencies have approved UCB-HSCs to treat a broad range of hematological, genetic, and immunological diseases, including acute and chronic leukemia, lymphomas, thalassemia, sickle cell disease, and congenital immunodeficiency [62–64].

Their regenerative potential and lower risk of graft-versus-host disease (GvHD), even in partially HLA-mismatched transplants, account for the clinical success of UCB-HSCs. The immunological immaturity of neonatal immune cells contributes to this lower risk [65].

In addition to hematopoietic applications, UCB-derived CD34⁺ cells and MSCs have shown promise in regenerative medicine for their angiogenic and neurotrophic properties. Ex vivo expansion of CD34⁺ cells can enhance the secretion of vascular and neural growth factors, suggesting additional therapeutic avenues in neurodegenerative and ischemic diseases [59, 64].

Moreover, UCB cells are a versatile source of cells for advanced therapeutic applications beyond traditional hematopoietic transplantation. Researchers are currently exploring UCB-derived immune cells in a range of experimental cellular therapies, including the generation of Chimeric Antigen (CAR) T cells, regulatory T cells, virus-specific T cells, and NK cells. These approaches primarily enhance immune responses in immunocompromised patients and treat viral infections [66].

UCB-MSCs exhibit a fibroblast-like morphology and are multipotent with the ability to differentiate into osteoblasts, chondrocytes, and adipocytes. Immunophenotypically, they express CD73, CD90, and CD105 while lacking hematopoietic markers [67].

These cells exhibit enhanced biological properties compared to adult-derived MSCs, including a higher proliferative capacity, greater self-renewal potential, and maintenance of chromosomal stability after cryopreservation. They are also characterized by low immunogenicity, partly due to downregulated HLA expression, and secrete key immunomodulatory molecules such as IL-10, TGF-β, and PGE₂. Furthermore, UCB-MSCs possess robust homing capabilities, mediated by chemokine receptors and specific signals, allowing them to migrate toward sites of tissue injury and inflammation [68].

UCB-MSCs have been successfully employed in numerous clinical and preclinical settings. Notably, several studies have demonstrated their efficacy in articular cartilage regeneration and skin regeneration. In dermatology and wound healing, UCB-MSCs and their secretome—including exosomes—have demonstrated regenerative capabilities. These effects are primarily mediated through modulation of the TGF-β signaling pathway, resulting in accelerated wound closure, reduced inflammation, and improved skin architecture [69, 70].

Intra-articular injection of UCB-MSCs in patients with knee osteoarthritis has led to significant improvements in cartilage integrity, pain reduction, and overall joint function, with a strong safety profile [71, 72].

In pulmonary medicine, a recent 2025 clinical trial reported that patients with pulmonary fibrosis tolerated nebulized EVs derived from UCB-MSCs well showed improvements in respiratory function and quality of life [73]. In patients with COVID-19-induced acute respiratory distress syndrome, UCB-MSCs have been used to mitigate the hyper inflammatory “cytokine storm”, resulting in reduced systemic inflammation and favorable safety outcomes in early-phase clinical trials (Supplementary Table 1) [74].

Ongoing studies are examining UCB-MSCs for the treatment of autoimmune diseases, endocrine and metabolic disorders, and neurological conditions, as well asfor applications in regenerative dermatology [75].

**Fig. 4:**
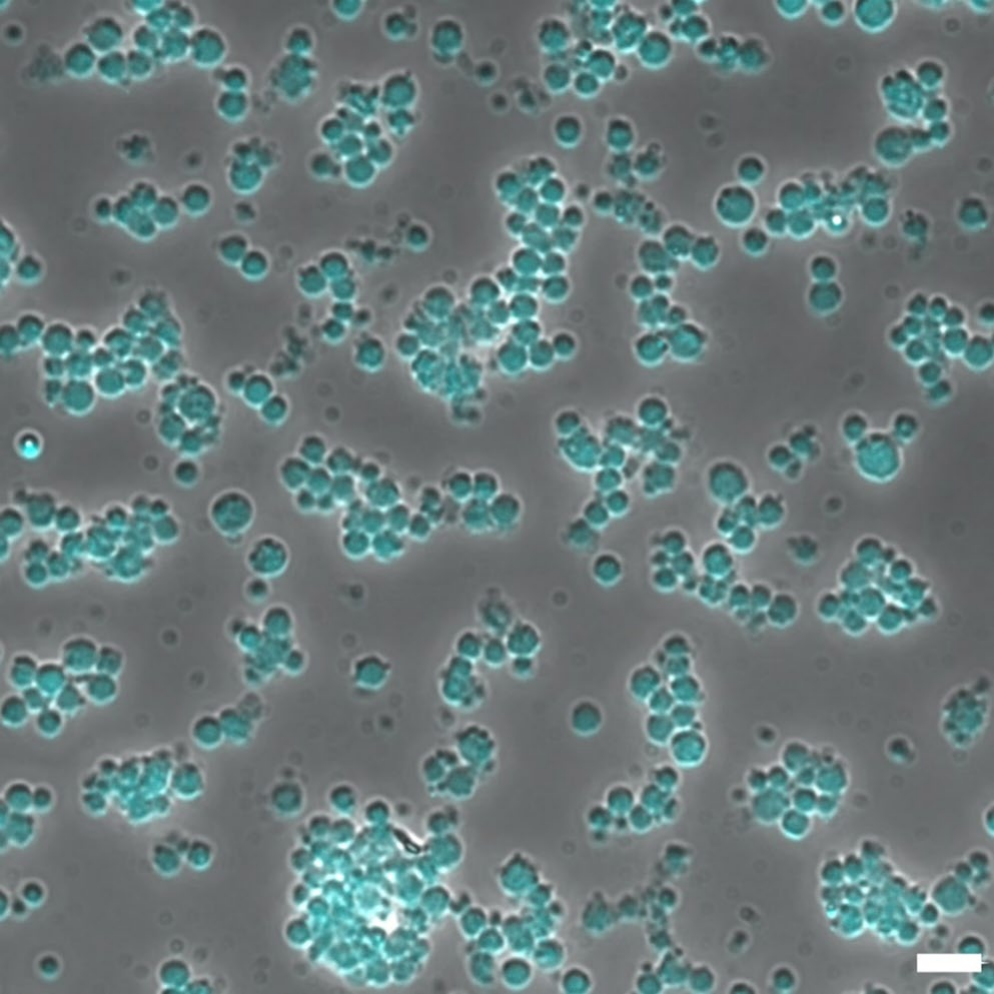
Phase contrast image of human UCB-derived VSEL. Nuclei are stained in blue (DAPI). Magnification: 180X, bar: 10 μm [78]

Finally, a rare and particularly intriguing population within UCB is that of Very Small Embryonic-Like cells (VSELs). These cells are significantly smaller than red blood cells (3–6 μm) and exhibit a high nucleus-to-cytoplasm ratio with undifferentiated chromatin (Fig. 4).

VSELs express pluripotency-associated markers such as OCT4, NANOG, and SSEA-4, and differentiate into the three germ layers under appropriate culture conditions. Evidences suggest that VSELs participate in neonatal tissue repair and fetomaternal chimerism [76–79]. However, they represent a promising but still controversial stem cell population [76–82]. Since their discovery, numerous independent studies challenge both the existence and functional significance of VSELs. Critics argue that isolation protocols may mistakenly sort cell debris, apoptotic bodies, or erythroblasts rather than living stem cells [80–82]. On the other hand, this may be due to imprecise selection methods, as VSELs are extremely small and rare (0.01%–0.03% of nucleated cells in bone marrow/UCB) [76, 83, 84].

Recent findings involving ex vivo cell expansion lay the foundations for potential applications in regenerative medicine [85]. Advancing isolation techniques and clarifying mechanisms of action, position UCB-derived VSELs as a promising pluripotent cell source for clinical therapies, particularly non-embryonic and ethically acceptable frameworks.

Despite the expanding clinical utility of UCB-derived cells, several limitations persist. A primary constraint is the limited volume of cord blood collected at birth, which often yields an insufficient number of cells—particularly hematopoietic or mesenchymal—for adult patients without ex vivo expansion. Moreover, MSCs occur at low frequencies in UCB, necessitating prolonged and labor-intensive culture protocols to achieve therapeutic doses.

Nonetheless, ongoing advances in cellular characterization, expansion techniques, and standardized isolation methods are progressively overcoming these barriers. These developments continue to enhance the scalability, consistency, and clinical impact of UCB-derived cell therapies, positioning them at the forefront of regenerative and personalized medicine.

#### Fetal Annexes: Amniotic Fluid and Placenta

 The placenta and amniotic fluid are vital fetal structures essential for intrauterine development and represent promising, ethically accepted sources of perinatal stem cells. The placenta is a transient organ formed from both fetal and maternal tissues. It serves as the primary interface between the mother and fetus, mediating nutritional, gaseous, hormonal, and immune exchanges. It can be collected safely and non-invasively after delivery, via either vaginal birth or cesarean section, making it a readily available and ethically favorable biological resource. In contrast, amniotic fluid surrounds the fetus during pregnancy, playing protective, antibacterial, and nutritional roles [86]. Its collection requires an invasive procedure (amniocentesis), typically performed between the 15th and 20th weeks of gestation for diagnostic purposes. Despite their differences, both sources harbor perinatal stem cells with highly favorable biological characteristics: low immunogenicity, absence of tumorigenicity, notable plasticity, and production of bioactive factors with regenerative effects.

Specifically, the placenta consists of three main populations [87–89]:

- Human Amniotic Epithelial Stem Cells (hA-ESCs), derived from the inner membrane of the placenta (the amnion), are particularly compelling due to their high plasticity, expression of pluripotency-associated markers, and low immunogenicity and tumorigenicity. These cells also release pro-regenerative factors with potential in wound healing, neuroregeneration, and epithelial repair [87, 90].
- Human Amniotic Mesenchymal Stem Cells (hA-MSCs), derived from the amniotic mesenchyme, are multipotent cells that share similarities with adult MSCs, including the ability to differentiate into osteogenic, chondrogenic, and adipogenic lineages. They secrete cytokines, exosomes, and exhibit strong immunomodulatory capacity [91].
- Mesenchymal Stromal Cells from the Amniochorionic Membrane (hFM-MSCs), originated from the fetal side of the placental wall, express key pluripotency genes, such as OCT4, NANOG, and SOX2. They are capable of mesodermal differentiation and contribute to bone regeneration and immune modulation [92].

Instead, the amniotic fluid contains Amniotic Fluid Stem Cells (AFSCs), which possess characteristics intermediate between embryonic and adult stem cells. They exhibit variable morphology (round or elongated), express embryonic markers such as SSEA-4, OCT4, and CD90, and show low tumorigenicity. Functionally, they can differentiate into cardiac, hepatic, neuronal, and pancreatic cells, and release bioactive exosomes and microRNAs with therapeutic relevance [91, 93].

Thanks to the properties described above, stem cells derived from the placenta and amniotic fluid have demonstrated considerable promise in preclinical and early clinical settings across multiple organ systems. For example, placental MSCs have shown efficacy in treating neonatal pulmonary disorders, such as bronchopulmonary dysplasia, by reducing inflammation and improving oxygenation [94]; in promoting intestinal mucosa regeneration in preclinical models of inflammatory bowel disease [95], and in stimulating neuronal regeneration following injury in rodents [96]. A 2023 review further highlights how placental cells, exosomes, and decellularized matrices can be integrated with advanced biomaterials and tissue engineering techniques (such as 3D bio printing and composite scaffolds) to regenerate skin, bone, cartilage, and the cardiovascular system, positioning the placenta as a “bio-factory” for multi-organ regenerative therapies [97]. hA-MSCs demonstrate significant regenerative potential across several medical fields. In the cardiovascular system, these cells reduce fibrosis and limit necrotic tissue following myocardial infarction [98]. In the pulmonary domain, amniotic fluid-derived mesenchymal stem cells effectively reduce inflammation and enhance alveolar permeability, improving lung function in preclinical models [99]. Moreover, in the musculoskeletal system, extracellular vesicles released by these stem cells counteract steroid-induced osteoporosis in vitro [100]. Despite this remarkable potential, the clinical use of these sources remains limited by regulatory challenges, the variability of isolation protocols, and the need for further safety and efficacy validation in human studies.

#### Wharton’s Jelly

Wharton’s jelly is a gelatinous connective tissue derived from the extraembryonic mesoderm, surrounding the umbilical cord vessels (two arteries and one vein). Rich in collagen, hyaluronic acid, proteoglycans, and glycoproteins, its primary role is to protect umbilical vessels from mechanical compression [101]. It has gained attention as a biological waste source of MSCs (WJ-MSCs, Wharton’s jelly MSCs), which resemble bone marrow and adipose-derived MSCs but may possess superior plasticity and proliferation capacity [102].

WJ-MSCs can be isolated via enzymatic digestion or explant culture [101]. They express typical MSCs markers (CD105, CD73, and CD90) and pluripotency markers like OCT4, NANOG, and SOX2. These multipotent cells differentiate into osteogenic, chondrogenic, adipogenic lineages, as well as mesodermal-derived cells such as neurons and pancreatic cells under appropriate induction [103].

Growing interest has also focused on the secretome of WJ-MSCs, which includes soluble factors (cytokines, chemokines, growth factors, and enzymes) as well as EVs (exosomes and microvesicles), which hold therapeutic promise in “cell-free” approaches [104] WJ-MSCs immunomodulatory properties support their use in autoimmune disease (e.g., lupus erythematosus and multiple sclerosis) and graft-versus-host disease (GVHD) by inhibiting the activation of T and B-lymphocytes, NK cells, and macrophages [104]. They promote liver regeneration by reducing fibrosis in chronic hepatitis and cirrhosis models [105], enhance cardiac repair post-myocardial infarction [106], and provide neuroprotection by secreting anti-inflammatory molecules [107]. Other promising applications include renal and lung repair, chronic wound healing and support for pancreatic function in diabetes [106].

In experimental oncology, EVs from WJ-MSCs carrying microRNAs, such as miR-125b, can inhibit tumor angiogenesis and proliferation in triple-negative breast cancer models [108].

Despite these promising perspectives, the clinical use of WJ-MSCs and their secretome is hindered by donor variability, in vitro senescence, challenges in standardizing dosing and delivery, and heterogeneity in secretome composition and isolation protocols [104].

#### Menstrual Blood

 Menstrual blood is a complex biological fluid composed of blood, cervical mucus, shed endometrial epithelial cells, immune cells, red blood cells, platelets, occasional genital tract cells, and vaginal secretions, cyclically expelled during menstruation. It originates from the shedding of the functional layer triggered by the drop in progesterone and estrogen in the absence of fertilization. Historically considered waste, menstrual blood is now recognized as a rich, non-invasive source of adult stem cells with regenerative potential [16, 109, 110]. 

The most notable are menstrual blood-derived stem cells (MenSCs), multipotent adult stem cells similar to bone marrow MSCs. In culture, they initially display radial or helical growth, followed by a spindle-shaped morphology, and expand rapidly with genomic stability and no tumorigenic or immunogenic risks [111]. MenSCs express MSC markers CD29, CD44, CD73, CD105, HLA-ABC, and lack hematopoietic markers [112]. Interestingly, they also express pluripotency markers (OCT4, SOX2, c-Myc, and NANOG), although the expression of c-Kit and SSEA-4 remains uncertain [112–114]. These cells can differentiate into osteogenic, chondrogenic, and adipogenic lineages, albeit with lower osteo/adipogenic potential than bone marrow MSCs [114]. They also differentiate into various other cell types, including germ cells, endometrial, endothelial, cardiomyocyte, respiratory epithelial, neural, muscle, hepatic, and pancreatic cells [115].

MenSCs can differentiate into female reproductive cells. In vitro, they acquire ovarian-like features expressing FSHR, LHR, and oocyte-related genes, including STRA8, GDF9, SCP3, and DDX4, which suggests their applicability in reproductive regenerative medicine [116]. Animal and early clinical studies demonstrate that MenSCs can regenerate ovarian function in premature ovarian insufficiency, thereby improving endocrine and menstrual parameters [115, 117].

MenSCs secrete cytokines with antifibrotic, angiogenic, anti-inflammatory, and immunoregulatory properties. In a recent murine model of liver fibrosis, MenSCs reduce fibrosis via ECM1 protein secretion acting on FoxO1 and mTOR pathways [118]; they have also shown positive therapeutic potential for the treatment of various cardiovascular diseases [119], while in respiratory diseases like acute respiratory distress syndrome, cryopreserved MenSCs restore alveolar structure, reduce inflammation, and improve gas exchange [120]. For musculoskeletal repair, MenSCs embedded in a collagen gel preserve the intervertebral disc structure after injury, as shown in a rat model [121].

Alongside cell-based applications, growing interest has emerged in the cell-free use of MenSCs, their secretome, and EVs. MenSC-derived exosomes show promising effects in reducing granulosa cell apoptosis in chemotherapy-induced ovarian damage models [122]. They have also proven effective in models of fulminant hepatic failure by improving liver function, enhancing survival, and reducing hepatocyte apoptosis [123]. Furthermore, in murine models of endometriosis, these exosomes inhibited lesion growth, angiogenesis, cellular proliferation, and invasiveness [124].

Despite their promise, MenSCs face challenges, including limited sample volume, donor variability, and a lack of standardized protocols for isolation and clinical use. Most evidence remains preclinical, with long-term efficacy and safety yet to be firmly established.

#### Dental Pulp

Dental pulp is a loose connective tissue that is highly vascularized and innervated. It resides in the inner central cavity of the tooth, closely associated with dentin, and together they form the dentin–pulp complex. This tissue consists mainly of two zones: the central zone, rich in blood vessels and nerves, and the peripheral zone, which contains odontoblasts that produce dentin. Unlike other connective tissues, dental pulp occupies a rigid cavity and receives limited vascular access only through the root apex [125].

The dental pulp performs multiple essential functions, including formation of dentin, nourishment of the tooth, protection from mechanical insults through the production of secondary dentin, and the transmission of sensitivity via nerve fibers responsive to thermal and mechanical stimuli [126].

Dental pulp collection involves the use of naturally exfoliated deciduous teeth or extracted permanent teeth, thereby maintaining a minimally invasive process.

This tissue has a heterogeneous composition: as mentioned, its outermost layer includes odontoblasts. Beneath them lies the acellular zone of Weil, rich in unmyelinated nerve fibers, capillaries, and fibroblastic processes. The main component is the extracellular matrix, also referred to as the “cell-rich zone” or “pulp core” [125]. Several cell types reside within dental pulp, including: fibroblasts, the most abundant cells; immune cells, present even under physiological conditions, such as granulocytes, T lymphocytes, monocytes/macrophages, dendritic cells, and to a lesser extent, NK cells, B cells, and regulatory T cells [126]. The central region, the most densely populated area, contains dental pulp stem cells (DPSCs) [18, 127]. Extraction of pulp tissue from the tooth’s internal chamber, following disinfection and longitudinal tooth opening, yields DPSCs. Immediately processing or cryopreservation preserves the tissue for later use. Enzymatic digestion with collagenase type I and dispase produces a cell suspension for standard culture conditions. Plastic adherence and fibroblast-like morphology identify stem cells, which then expand in culture for research or therapy [128].

DPSCs are mesenchymal progenitor cells mainly derived from the cranial neural crest. In addition to expressing typical mesenchymal markers, (CD29, CD44, CD73, CD90, CD105, CD146, Stro-1, CD13), DPSCs also exhibit positivity for pluripotency markers (OCT4, NANOG, SOX2, SSEA-3, SSEA-4, TRA-1–60, TRA-1–81), odontoblastic markers (Dentin sialophosphoprotein – DSPP; Dentin Matrix Protein-1 – DMP-1), and neural stem cell markers such as GFAP, Nestin, P75, HNK-1, and S-100. They are negative for hematopoietic markers including CD34, CD45, CD14, CD11b, and HLA-DR [18, 129–131].

**Fig. 5:**
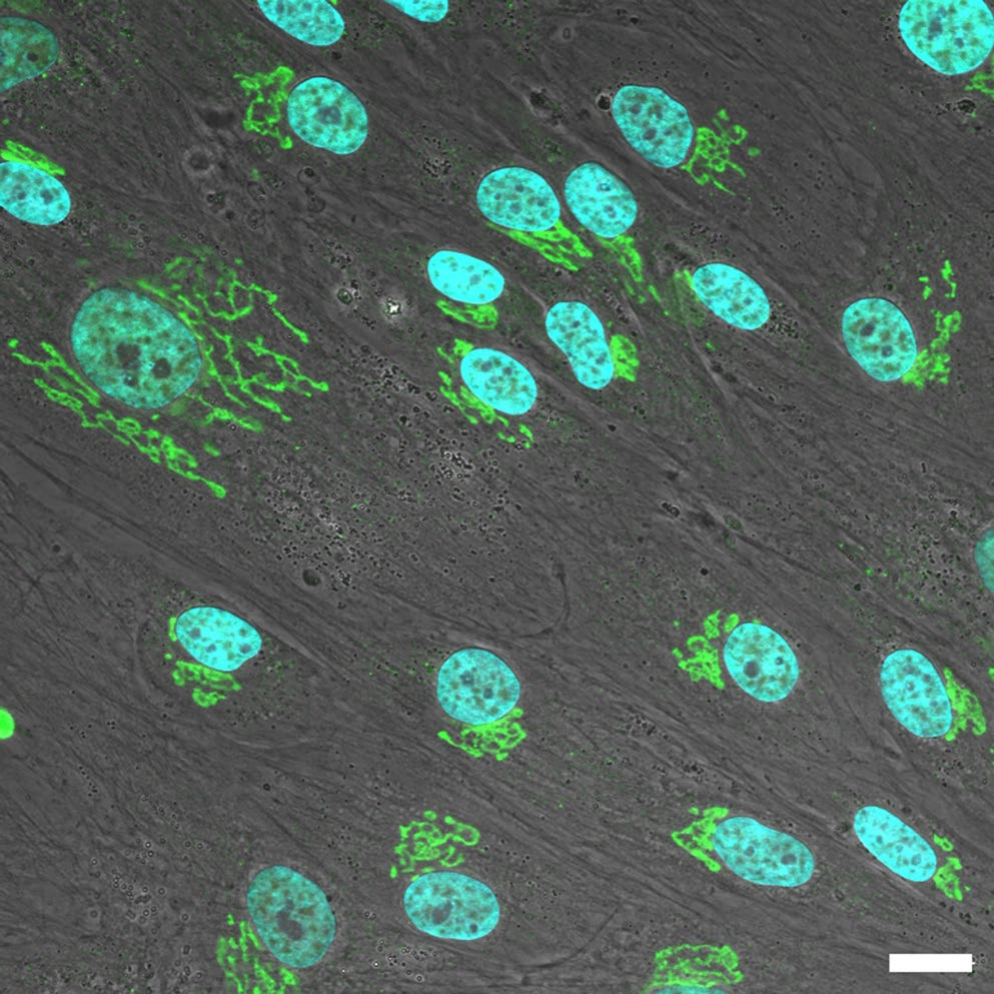
Phase contrast image of human DPSCs. Nuclei are stained in blue (DAPI), Golgi apparatus in green. Magnification: 60X, bar: 10 μm [128]

In vitro, DPSCs show high proliferative capacity, colony formation ability, superior replicative potential, and genomic stability compared to bone marrow-derived MSCs [129, 131]. They can differentiate into odontoblast-like cells forming mineralized nodules and dentin-like structures, as well as into osteogenic, chondrogenic, adipogenic, neurogenic, myogenic, endothelial, and pancreatic lineages [128, 131, 132] (Fig. 5).

 In dentistry, combinations of DPSCs and scaffolds regenerate functional, vascularized pulp-like tissues, and marksignificant progress in regenerative endodontic therapy [133–135]. Additionally, DPSCs exhibit strong osteogenic potential and have been applied in mandibular and craniofacial bone regeneration, demonstrating enhanced vascularization and bone formation compared to acellular scaffolds [136].

DPSCs have contributed to functional recovery in preclinical spinal cord injury models by expressing neuronal markers and secreting neurotrophic factors (NGF, BDNF), supporting axonal regeneration and reducing inflammation [137, 138]. They also promote angiogenesis and aid tissue regeneration in ischemic conditions.

Furthermore, DPSCs accelerate wound healing by secreting anti-inflammatory and pro-regenerative factors, thereby providing therapeutic benefits for chronic wounds and ulcers [139, 140]. They have also demonstrated potential in smooth muscle regeneration, such as bladder reconstruction, and exhibit immunomodulatory properties relevant to the treatment of autoimmune and metabolic diseases [141–143].

Despite these promising preclinical and clinical applications, DPSCs face challenges for large-scale use: limited tissue volume and low cell yield per tooth, biological properties that vary with donor age, with deciduous tooth-derived DPSCs exhibiting higher proliferative capacity than those from adult teeth, and a lack of large-scale clinical trials to validate their safety and efficacy in humans (Supplementary Table 1).

#### Regulatory Considerations for the Use of Stem Cells Derived from Biological Waste Materials

Stem cells from biological waste materials offer a promising alternative to embryonic sources. These materials raise fewer ethical concerns. However, regulatory bodies still enforce complex oversight to address the remaining ethical issues and ensure proper governance. Such oversight protects donor rights, ensures product quality, and supports the safe and effective use of these cell products in clinical settings.

#### Regulatory Pathways

**Fig. 6:**
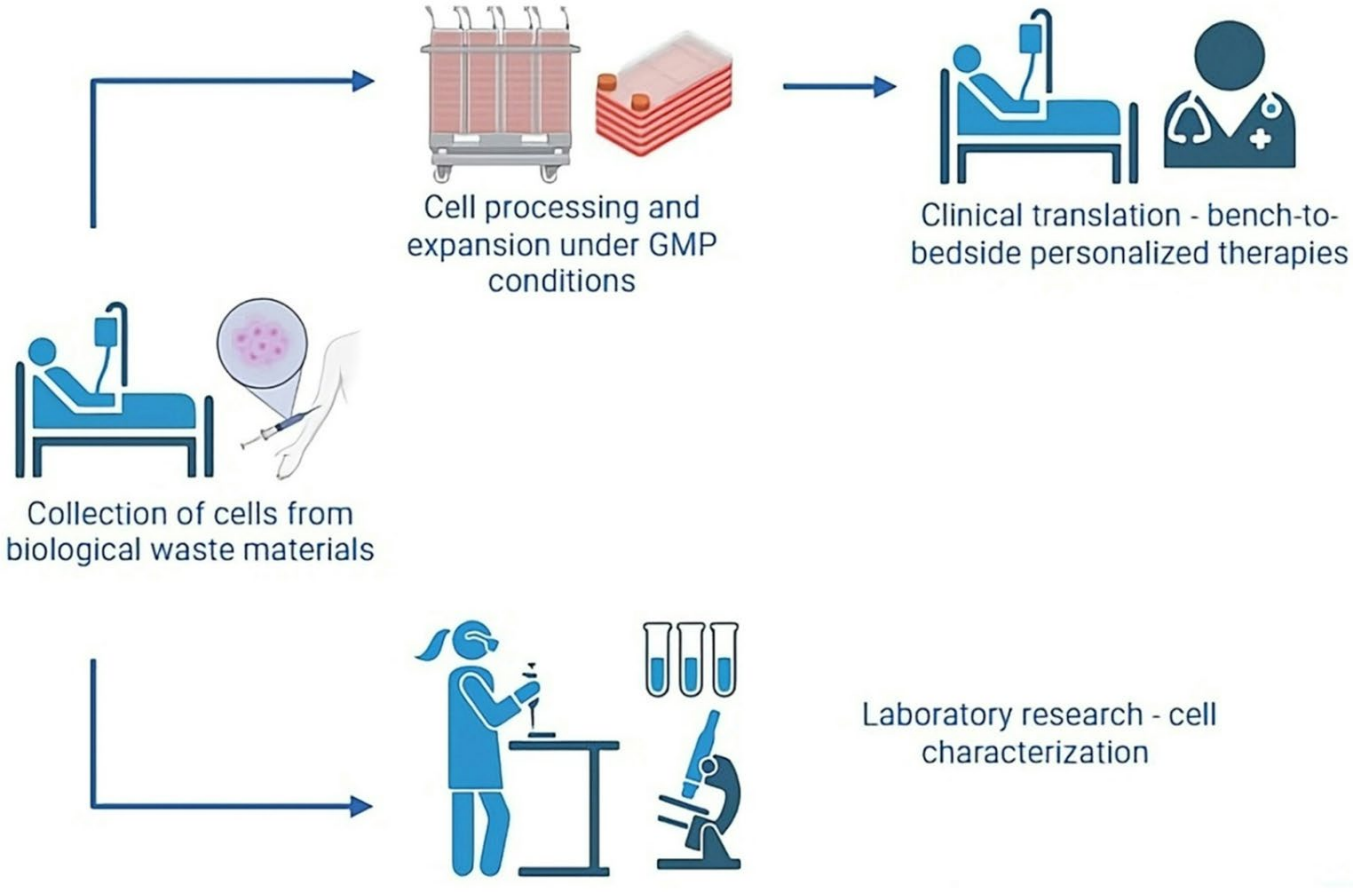
The regulatory pipeline encompasses patient-derived sample collection, GMP (Good Manufacturing Practice)- compliant cell processing, laboratory characterization, and ultimately, clinical translation through personalized therapies that span the bench-to-bedside continuum. Created in BioRender.com

The extent of manipulation directly shapes manufacturing requirements. Researchers who process cells extensively must use Good Manufacturing Practice (GMP) conditions. This maintains sterility, identity, potency, and reproducibility. Laboratory interventions introduce variability and potential risks, so stringent quality controls are required. Investigators must rigorously screen waste-derived tissues for infectious agents, genetic abnormalities, and potential contaminants. Long-term safety risks, including immunogenicity, genomic instability, and tumorigenicity must also be assessed. Preclinical and clinical testing frameworks require comprehensive data. Regulators demand robust, long-term safety evidence before approving therapeutic applications (Fig. 6).

Countries worldwide have established regulatory frameworks to oversee the development and clinical use of gene- and cell-based therapies. Safety, efficacy, and quality assurance are the core principles [144, 145].

- United States: the Food and Drug Administration (FDA) regulates these materials as Human Cells, Tissues, and Cellular and Tissue-Based Products (HCT/Ps) under 21 CFR Part 1271. Specifically, regulators primarily determine classification by the degree of manipulation. Products that undergo only “minimal manipulation” and serve homologous use may qualify for less burdensome oversight. In contrast, products that undergo extensive manipulation (e.g., expansion, differentiation, or genetic engineering) fall under the category of biological products and require premarket approval.
- Canada regulates gene and cell therapies as biologics under the Food and Drugs Act and its accompanying regulations, including the Food and Drug Regulations and the Safety of Human Cells, Tissues and Organs for Transplantation Regulations. Cell-based therapies generally involve processing a patient's cells. Gene therapies aim to modify a patient's genetic material to treat conditions. Classification depends on intended use, extent of modification, and associated risks. Health Canada established the Advanced Therapeutic Goods pathway for new products or those that do not easily fit into the existing categories.
- The European Union: The European Medicines Agency (EMA) applies the Advanced Therapy Medicinal Products (ATMP) framework regulated by the EU Directive 2001/83/EC on ATMPs, which also distinguishes between processing levels and therapeutic intent. The EMA provides also provides centralized marketing authorization across EU member states. Programs such as PRIME (PRIority Medicines) and Conditional Marketing Authorization expedite access for therapies targeting severe or rare diseases, while orphan drug incentives encourage innovation in rare disease therapeutics.
- Japan: the Pharmaceuticals Medical Devices Act (PMD Act) regulates cell therapy products as regenerative medicine products. Japan has pioneered progressive regulation through the Act on the Safety of Regenerative Medicine and the Sakigake designation, which enable conditional, time-limited approvals coupled with rigorous post-marketing surveillance. These frameworks establish Japan as a leader in regenerative medicine.
- China: Since 2017, the National Medical Program Administration has reformed China’s approval processes. Regulations ensure GMP compliance and ethical oversight of stem cell research.
- India: The Central Drugs Standard Control Organization (CDSCO) is the national regulatory body. It ensures the safety, efficacy, and quality of drugs, cosmetics, and medical devices. Under the Drugs and Cosmetics Act, CDSCO approves new drugs and clinical trials, regulating drug imports, and setting quality standards.
- Australia: The Therapeutic Goods Administration (TGA) regulates therapies under the Biologicals Regulatory Framework, classifying products based on their risk level. Highly manipulated products face stringent pre-market evaluation. The Gene Technology Act and the National Health and Medical Research Council (NHMRC) ethical guidelines ensure the safe development of genetically modified therapies and ethical conduct in clinical trials.
- Brazil: ANVISA (Brazilian health regulatory agency) regulates ATMPs under RDC No. 260/2018. This covers somatic cell therapy, gene therapy, and tissue engineering. Oversight focuses on manipulation level, cell source, and therapeutic intent, requiring strict adherence to safety, efficacy, and quality standards prior to approval.

Overall, despite national variations, regulatory frameworks share a common foundation of ensuring safety, efficacy, and quality. They also provide accelerated pathways and incentives to encourage innovation. Japan and China emphasize conditional approvals with post-market monitoring, while the United States and Europe focus on rigorous pre-market evaluation and expedited designations. These complementary approaches collectively advance global progress in regenerative medicine.

#### Ethical and Legal Dimensions

 Although waste-derived stem cells circumvent most ethical controversies linked to embryonic stem cell research. Some unresolved issues remain. This include commercialization, donor rights, and equitable access to therapies. In many countries, legislative restrictions on the ownership, transfer, and commercialization of biological waste hinder cross-border collaboration and delay clinical translation.

#### Efforts to Harmonize International Standards

 As gene- and cell-based therapies gain global prominence, regulatory authorities and international organizations increasingly seek to harmonize standards for ATMPs. Harmonization aims to reduce duplication, streamline development, and ensure consistent safety, efficacy, and quality across jurisdictions. International initiatives, such as guidelines from the International Council for Harmonization of Technical Requirements for Pharmaceuticals for Human Use (ICH) and recommendations from the International Society for Stem Cell Research (ISSCR), provide frameworks for ethical conduct, preclinical testing, and clinical evaluation. Regulatory agencies are aligning definitions such as “minimal manipulation” and “homologous use,” facilitating cross-border clinical trials and technology transfer. Accelerated pathways, such as the FDA’s Breakthrough Therapy designation, the EMA’s PRIME scheme, and Japan’s Sakigake designation, expedite the approval of new therapies. These programs maintain rigorous oversight. Harmonized guidelines emphasize GMP-compliant manufacturing, robust potency and identity testing, and strict safety monitoring, including standardized adverse event reporting and post-market surveillance. Despite these advances, differences in legal frameworks, national priorities, and resource levels remain. These variations result in regulatory fragmentation. Ongoing dialogue among regulators, industry, and academic stakeholders aims to establish mutual recognition agreements, standardized terminology, and unified clinical trial protocols. This will accelerate the translation of ATMPs from research to clinical use while safeguarding patient safety and scientific integrity (https://www.ich.org/; https://www.isscr.org/).

## Conclusions and Future Challenges

 The use of biological waste materials in research and health sectors is central to advancing scientific progress and improving healthcare outcomes. These materials provide a foundation for developing new medical treatments and expanding our understanding of diseases. They also drive biotechnological innovation. As a result, they directly shape cost-effective research and enable breakthroughs in regenerative medicine, drug development, and diagnostic technologies [146].

Building on these promising applications, the translation of stem cells derived from biological waste into therapeutic products necessitates the establishment and maintenance of GMP-compliant facilities. These facilities present significant financial barriers. Production costs per patient dose vary according to cell type, scalability, and the extent of cell manipulation. The primary expenses stem from requirements for sterile infrastructure, rigorous quality control testing, validated processing protocols, and long-term product tracking and monitoring.

To address these challenges, artificial intelligence (AI) is emerging as a transformative tool in optimizing manufacturing protocols for advanced therapies, including stem cell–derived products and other ATMPs. Machine learning algorithms can analyze large datasets generated during cell culture, expansion, and differentiation. They can identify optimal growth conditions and predict batch outcomes. For example, deep learning models applied to imaging data can non-invasively monitor cell morphology and function, reducing reliance on destructive assays while ensuring reproducibility. Similarly, reinforcement learning and digital twin technologies enable the simulation of bioprocess parameters, including nutrient delivery, oxygenation, and shear stress, before implementation. As a result, these tools reduce the experimental burden and the likelihood of manufacturing failures.

Within this context, in GMP settings, AI can support closed-system automation by integrating with robotics and advanced sensors. These systems maintain consistent environmental conditions and minimize human error. They also ensure compliance with regulatory standards. Importantly, AI-powered predictive analytics also enhance supply chain management and cost modeling. This offers the potential to lower the cost of goods sold (COGS), which remains a key barrier to the large-scale clinical translation of ATMPs.

Nevertheless, despite its promise, the integration of AI into manufacturing protocols faces regulatory and ethical challenges. Both the EMA and FDA emphasize the need for transparency, validation, and explainability in AI-driven decision-making. This is necessary to ensure patient safety and regulatory compliance. Harmonized guidelines on AI in ATMP manufacturing are still developing. However, pilot projects suggest that AI-enabled automation can accelerate scale-up while maintaining stringent quality assurance.

In summary, adopting AI in ATMP manufacturing is critical for optimizing processes, reducing variability, and lowering costs. As regulatory frameworks adapt to digital technologies, AI will become integral to ensuring scalable and reliable cell-based therapies, establishing it as a linchpin for future advancements in the field.

The strategic use of biological waste materials is crucial for advancing health research, but it demands careful navigation of legal and ethical complexities. Establishing harmonized frameworks and ethical standards is crucial not only for maximizing benefits but also for supporting responsible and equitable progress in the field.

## Supplementary Information

Below is the link to the electronic supplementary material.

Supplementary Material 1 (DOCX 26.4 KB)

## Data Availability

No datasets were generated or analysed during the current study.

## References

[1] Elizondo-Omaña, R. E., Guzmán-López, S., & García-Rodríguez, M. L. (2005). Dissection as a teaching tool: past, present, and future. *The Anatomical Record Part B: the New Anatomist*, *285*(1), 11-5. 10.1002/ar.b.20070. 10.1002/ar.b.20070 16032753

[2] van Tellingen, C. (2007). Pliny’s pharmacopoeia or the Roman treat. *Netherlands Heart Journal,**15*(3), 118. 10.1007/BF03085966 18604277 10.1007/BF03085966 PMC2442893

[3] Prioreschi, A. (1991). *History of medicine* (pp. 182–211). F. Norman Library of Science and Medicine No. 424. Hook & Norman, The Haskell.

[4] Ghosh, S. K. (2015). Human cadaveric dissection: A historical account from ancient Greece to the modern era. *Anatomy and Cell Biology,**48*(3), 153–69. 10.5115/acb.2015.48.3.153 26417475 10.5115/acb.2015.48.3.153 PMC4582158

[5] Kemp, M. (2006). *Leonardo Da vinci: The marvellous works of nature and man*. Oxford University Press. 10.1136/jmh.2008.000275 23674544

[6] Cunningham, A. (1997). *The anatomist anatomis’d: An experimental discipline in enlightenment Europe*. Routledge.

[7] Skloot, R. (2010). *The immortal life of Henrietta lacks*. Crown Publishing Group.

[8] Huang, Y., Wu, Q., & Tam, P. K. H. (2023). Immunomodulatory mechanisms of mesenchymal stem cells and their potential clinical applications. *International Journal of Molecular Sciences*, *23*(17), 10023. 10.3390/ijms231710023 10.3390/ijms231710023 PMC9456387 36077421

[9] Alnasser, S. M., Alrobian, A. S., Alfayez, M. S., Almutairi, O. T., Almutairi, S. S., & Alkeraidees, T. S. (2025). Pharmacological modulation of stem cells signaling pathway for therapeutic applications. *Stem Cell Research & Therapy*, *16*(1), 327. 10.1186/s13287-025-04438-8 40598543 10.1186/s13287-025-04438-8 PMC12210473

[10] Tian, Z., Yu, T., Liu, J., Wang, T., & Higuchi, A. (2023). Introduction to stem cells. *Progress in Molecular Biology and Translational Science*, *199*, 3–32. 10.1016/bs.pmbts.2023.02.012 37678976 10.1016/bs.pmbts.2023.02.012

[11] Takahashi, K., Tanabe, K., Ohnuki, M., Narita, M., Ichisaka, T., Tomoda, K., & Yamanaka, S. (2007). Induction of pluripotent stem cells from adult human fibroblasts by defined factors. *Cell*, 131(5),861 − 72. 10.1016/j.cell.2007.11.019. 10.1016/j.cell.2007.11.019 18035408

[12] Ratajczak, M. Z., Zuba-Surma, E., Kucia, M., Poniewierska, A., Suszynska, M., & Ratajczak, J. (2012). Pluripotent and multipotent stem cells in adult tissues. *Advances in Medical Sciences,**57*(1), 1–17. 10.2478/v10039-012-0020-z 22515973 10.2478/v10039-012-0020-z

[13] Lennon, D. P., & Caplan, A. I. (2006). Isolation of human marrow-derived mesenchymal stem cells. *Experimental Hematology*, 34(11),1604-5. 10.1016/j.exphem.2006.07.014. 10.1016/j.exphem.2006.07.014 17046583

[14] Al-Ghadban, S., Artiles, M., & Bunnell, B. A. (2022). Adipose stem cells in regenerative medicine: Looking forward. *Frontiers in Bioengineering and Biotechnology,**9*, Article 837464. 10.3389/fbioe.2021.837464 35096804 10.3389/fbioe.2021.837464 PMC8792599

[15] Zhang, D., Wei, G., Li, P., Zhou, X., & Zhang, Y. (2014). Urine-derived stem cells: A novel and versatile progenitor source for cell-based therapy and regenerative medicine. *Genes & Diseases*, *1*(1), 8–17. 10.1016/j.gendis.2014.07.001 25411659 10.1016/j.gendis.2014.07.001 PMC4234168

[16] Bozorgmehr, M., Gurung, S., Darzi, S., Nikoo, S., Kazemnejad, S., Zarnani, A. H., & Gargett, C. E. (2020). Endometrial and menstrual blood mesenchymal stem/stromal cells: Biological properties and clinical application. *Frontiers in Cell and Developmental Biology,**8*, Article 497. 10.3389/fcell.2020.00497 32742977 10.3389/fcell.2020.00497 PMC7364758

[17] Weiss, M. L., & Troyer, D. L. (2006). Stem cells in the umbilical cord. *Stem Cell Reviews and Reports*, *2*(2),155-62. 10.1007/s12015-006-0022-y. 10.1007/s12015-006-0022-y PMC3753204 17237554

[18] Tatullo, M., Marrelli, M., Shakesheff, K. M., & White, L. J. (2015). Dental pulp stem cells: Function, isolation and applications in regenerative medicine. *Journal of Tissue Engineering and Regenerative Medicine*, *9*(11), 1205–1216. 10.1002/term.1899 24850632 10.1002/term.1899

[19] Urinology Think Tank Writing Group. (2018). Urine: Waste product or biologically active tissue? *Neurourology and Urodynamics*, *37*(3), 1162–1168. 10.1002/nau.23414 29464759 10.1002/nau.23414 PMC5897180

[20] Sun, Y., Zhao, H., Yang, S., Wang, G., Zhu, L., Sun, C., & An, Y. (2024). Urine-derived stem cells: Promising advancements and applications in regenerative medicine and beyond. *Heliyon,**10*(6), Article e27306. 10.1016/j.heliyon.2024.e27306 38509987 10.1016/j.heliyon.2024.e27306 PMC10951541

[21] Yu, P., Bosholm, C. C., Zhu, H., Duan, Z., Atala, A., & Zhang, Y. (2024). Beyond waste: Understanding urine’s potential in precision medicine. *Trends in Biotechnology,**42*(8), 953–969. 10.1016/j.tibtech.2024.01.009 38369434 10.1016/j.tibtech.2024.01.009 PMC11741143

[22] Zhang, Y., McNeill, E., Tian, H., Soker, S., Andersson, K. E., Yoo, J. J., & Atala, A. (2008). Urine derived cells are a potential source for urological tissue reconstruction. *Journal of Urology,**180*(5), 2226–2233. 10.1016/j.juro.2008.07.023 18804817 10.1016/j.juro.2008.07.023

[23] Manaph, P. A., Al-Hawwas, N., Bobrovskaya, M., Coates, L., P.T., & Zhou, X. F. (2018). Urine-derived cells for human cell therapy. *Stem Cell Research & Therapy*, *9*, 189. 10.1186/s13287-018-0932-z 29996911 10.1186/s13287-018-0932-z PMC6042455

[24] Tayhan, S. E., Keleş, G. T., Topçu, İ., Mir, E., & Gürhan, S. İ. D. (2017). Isolation And in vitro cultivation of human urine-derived cells: An alternative stem cell source. *Turkish Journal of Urology*, *43*(3), 345–349. 10.5152/tud.2017.93797 28861309 10.5152/tud.2017.93797 PMC5562256

[25] Boysen, A. T., Whitehead, B., Revenfeld, A. L. S., Gupta, D., Petersen, T., & Nejsum, P. (2024). Urine-derived stem cells serve as a robust platform for generating native or engineered extracellular vesicles. *Stem Cell Research & Therapy*, *15*(1), 288. 10.1186/s13287-024-03903-0 39256816 10.1186/s13287-024-03903-0 PMC11389316

[26] Bharadwaj, S., Liu, G., Shi, Y., Wu, R., Yang, B., He, T., Fan, Y., Lu, X., Zhou, X., Liu, H., Atala, A., Rohozinski, J., & Zhang, Y. (2013). Multipotential differentiation of human urine-derived stem cells: Potential for therapeutic applications in urology. *Stem Cells,**31*(9), 1840–1856. 10.1002/stem.1424 23666768 10.1002/stem.1424

[27] Bento, G., Shafigullina, A. K., Rizvanov, A. A., Sardão, V. A., Macedo, M. P., & Oliveira, P. J. (2020). Urine-derived stem cells: Applications in regenerative and predictive medicine. *Cells,**9*(3), Article 573. 10.3390/cells9030573 32121221 10.3390/cells9030573 PMC7140531

[28] Zhou, Q., Cheng, Y., Sun, F., Shen, J., Nasser, M. I., Zhu, P., Zhang, X., Li, Y., Yin, G., Wang, Y., Wu, X., & Zhao, M. (2022). A comprehensive review of the therapeutic value of urine-derived stem cells. *Frontiers in Genetics,**12*, Article 781597. 10.3389/fgene.2021.781597 35047009 10.3389/fgene.2021.781597 PMC8762167

[29] Jiang, Z. Z., Liu, Y. M., Niu, X., Yin, J. Y., Hu, B., Guo, S. C., Wang, Y., & Wang, N. S. (2016). Exosomes secreted by human Urine-Derived stem cells could prevent kidney complications from type I diabetes in rats. *Stem Cell Research & Therapy*, *7*, 24. 10.1186/s13287-016-0287-2 26852014 10.1186/s13287-016-0287-2 PMC4744390

[30] Kim, S. H., Lee, S. H., Jin, J. A., So, H. J., Lee, J. U., Ji, M. J., Kwon, E. J., Han, P. S., Lee, H. K., & Kang, T. W. (2023). In vivo safety and biodistribution profile of Klotho-enhanced human urine-derived stem cells for clinical application. *Stem Cell Research & Therapy*, *1*, 355. 10.1186/s13287-023-03595-y 10.1186/s13287-023-03595-y PMC10712141 38072946

[31] Zhou, T., Benda, C., Dunzinger, S., Huang, Y., Ho, J. C., Yang, J., Wang, Y., Zhang, Y., Zhuang, Q., Li, Y., Bao, X., Tse, H. F., Grillari, J., Grillari-Voglauer, R., Pei, D., & Esteban, M. A. (2012). Generation of human induced pluripotent stem cells from urine samples. *Nature Protocols,**7*(12), 2080–2089. 10.1038/nprot.2012.115 23138349 10.1038/nprot.2012.115

[32] Bispo, A. F. S., de Jesus Simao, J., Moraes, M. A., Abelm A,B,M., Plata, V. T. G., Telles, M. M., Santana, A. V., Volpe, P., Armelin-Correa, L. M., & Alonso-Vale, M. I. C. (2025). Effects of obesity-associated plasma markers on adipose stem cell function and epigenetic regulation. *Obesity (Silver Spring)*, *33*(8), 1529–1542. 10.1002/oby.24321 40518865 10.1002/oby.24321 PMC12304845

[33] Suchanecka, M., Grzelak, J., Farzaneh, M., Azizidoost, S., Dari, M. A. G., Józkowiak, M., Data, K., Domagała, D., Niebora, J., Kotrych, K., Czerny, B., Kamiński, A., Torlińska-Walkowiak, N., Bieniek, A., Szepietowski, J., Piotrowska-Kempisty, H., Dzięgiel, P., Mozdziak, P., & Kempisty, B. (2025). Adipose derived stem cells – Sources, differentiation capacity and a new target for reconstructive and regenerative medicine. *Biomedicine & Pharmacotherapy*. 10.1016/j.biopha.2025.118036. 186,118036. 10.1016/j.biopha.2025.118036 40194335

[34] Uguten, M., van der Sluis, N., Vriend, L., Coert, J. H., Harmsen, M. C., van der Lei, B., & van Dongen, J. A. (2024). Comparing mechanical and enzymatic isolation procedures to isolate adipose-derived stromal vascular fraction: A systematic review. *Wound Repair and Regeneration,**32*(6), 1008–1021. 10.1111/wrr.13228 39444305 10.1111/wrr.13228 PMC11584359

[35] Cremona, M., Gallazzi, M., Rusconi, G., Mariotta, L., Gola, M., & Soldati, G. (2025). State of the art in the standardization of stromal vascular fraction processing. *Biomolecules,**15*(2), Article 199. 10.3390/biom15020199 40001502 10.3390/biom15020199 PMC11852902

[36] Fu, H., & Wang, C. (2025). Micro-fragmented adipose tissue-an innovative therapeutic approach: A narrative review. *Medicine (Baltimore),**104*(9), Article e41724. 10.1097/MD.0000000000041724 40020111 10.1097/MD.0000000000041724 PMC11875617

[37] Jeyaraman, N., Shrivastava, S., Rangarajan, R. V., Nallakumarasamy, A., Ramasubramanian, S., Devadas, A. G., Rupert, S., & Jeyaraman, M. (2025). Challenges in the clinical translation of stromal vascular fraction therapy in regenerative medicine. *World Journal of Stem Cells,**17*(6), Article 103775. 10.4252/wjsc.v17.i6.103775 40585955 10.4252/wjsc.v17.i6.103775 PMC12203130

[38] Si, Z., Wang, X., Sun, C., Kang, Y., Xu, J., Wang, X., & Hui, Y. (2019). Adipose-derived stem cells: Sources, potency, and implications for regenerative therapies. *Biomedicine & Pharmacotherapy*. 10.1016/j.biopha.2019.108765 10.1016/j.biopha.2019.108765 30921703

[39] Mazini, L., Ezzoubi, M., & Malka, G. (2021). Overview of current adipose-derived stem cell (ADSCs) processing involved in therapeutic advancements: Flow chart and regulation updates before and after COVID-19. *Stem Cell Research & Therapy*, *12*, 1. 10.1186/s13287-020-02006-w 33397467 10.1186/s13287-020-02006-w PMC7781178

[40] Papadopoulos, K. S., Piperi, C., & Korkolopoulou, P. (2024). Clinical applications of Adipose-Derived stem cell (ADSC) exosomes in tissue regeneration. *International Journal of Molecular Sciences,**25*(11), Article 5916. 10.3390/ijms25115916 38892103 10.3390/ijms25115916 PMC11172884

[41] Mazini, L., Rochette, L., Amine, M., & Malka, G. (2019). Regenerative capacity of adipose derived stem cells (ADSCs), comparison with mesenchymal stem cells (MSCs). *International Journal of Molecular Sciences,**20*(10), Article 2523. 10.3390/ijms20102523 31121953 10.3390/ijms20102523 PMC6566837

[42] Foti, R., Storti, G., Palmesano, M., Scioli, M. G., Fiorelli, E., Terriaca, S., Cervelli, G., Kim, B. S., Orlandi, A., & Cervelli, V. (2024). Senescence in adipose-derived stem cells: Biological mechanisms and therapeutic challenges. *International Journal of Molecular Sciences,**25*(15), Article 8390. 10.3390/ijms25158390 39125960 10.3390/ijms25158390 PMC11312747

[43] Su, H., Chau, H., Li, Q., Xiao, F., Sun, Y., Chen, J., He, Y., Ning, J., Hu, Q., Xiao, Y., Li, C., Huang, B., Zhao, J., Li, Y., & Li, H. (2025). Bridging the gap: Clinical translation of adipose-derived stem cells - a scoping review of Clinical trials. *Stem Cell Research & Therapy*, *16*(1), 288. 10.1186/s13287-025-04405-3 40483503 10.1186/s13287-025-04405-3 PMC12145640

[44] Pan, Y., Pan, C., & Zhang, C. (2024). Unraveling the complexity of follicular fluid: Insights into its composition, function, and clinical implications. *Journal Of Ovarian Research,**17*(1), Article 237. 10.1186/s13048-024-01551-9 39593094 10.1186/s13048-024-01551-9 PMC11590415

[45] Zhang, Y., He, C., He, Y., & Zhu, Z. (2025). Follicular fluid metabolomics: Tool for predicting IVF outcomes of different infertility causes. *Reproductive Sciences,**32*(4), 921–934. 10.1007/s43032-024-01664-y 39090336 10.1007/s43032-024-01664-y PMC11978680

[46] Piccinni, M. P., Vicenti, R., Logiodice, F., Fabbri, R., Kullolli, O., Pallecchi, M., Paradisi, R., Danza, G., Macciocca, M., & Lombardelli, L. (2021). Seracchioli, R. Description of the follicular fluid cytokine and hormone profiles in human physiological natural cycles. *Journal of Clinical Endocrinology & Metabolism*, (2), e721–e738. 10.1210/clinem/dgaa880 10.1210/clinem/dgaa880 PMC7823236 33247906

[47] Rungsiwiwut, R., Numchaisrika, P., Thuwanut, P., & Pruksananonda, K. (2021). Characterization of stem cells from human ovarian follicular fluid; A potential source of autologous stem cell for cell-based therapy. *Human Cell,**34*, 300–309. 10.1007/s13577-020-00439-2 33543452 10.1007/s13577-020-00439-2

[48] Riva, F., Omes, C., Bassani, R., Nappi, R. E., Mazzini, G., Icaro Cornaglia, A., & Casasco, A. (2014). In-vitro culture system for mesenchymal progenitor cells derived from waste human ovarian follicular fluid. *Reproductive BioMedicine Online,**29*(4), 457–469. 10.1016/j.rbmo.2014.06.006 25131558 10.1016/j.rbmo.2014.06.006

[49] Fiscus, J., Fraison, É., Renault, L., Salle, B., Panthu, B., & Labrune, E. (2024). Metabolic signature of follicular fluid in infertility-related diseases: A narrative review. *Reproductive BioMedicine Online,**48*(6), Article 103762. 10.1016/j.rbmo.2023.103762 38537523 10.1016/j.rbmo.2023.103762

[50] Pla, I., Sanchez, A., Pors, S. E., Kristensen, S. G., Appelqvist, R., Sahlin, K. B., Marko-Varga, G., Andersen, C. Y., & Malm, J. (2022). Proteomic alterations in follicular fluid of human small antral follicles collected from polycystic ovaries—A pilot study. *Life,**12*(3), Article 391. 10.3390/life12030391 35330141 10.3390/life12030391 PMC8954146

[51] Kranc, W., Kaczmarek, M., Kowalska, K., Pieńkowski, W., Ciesiółka, S., Konwerska, A., Mozdziak, P., Brązert, M., Jeseta, M., Spaczyński, R. Z., Pawelczyk, L., & Kempisty, B. (2025). Morphological characteristics, extracellular vesicle structure and stem-like specificity of human follicular fluid cell subpopulation during osteodifferentiation. *Experimental And Molecular Pathology,**142*, Article 104965. 10.1016/j.yexmp.2025.104965 40253818 10.1016/j.yexmp.2025.104965

[52] Chandramohan, Y., Jeganathan, K., Sivanesan, S., Koka, P., Amritha, T. M. S., Vimalraj, S., & Dhanasekaran, A. (2021). Assessment of human ovarian follicular fluid derived mesenchymal stem cells in chitosan/PCL/Zn scaffold for bone tissue regeneration. *Life Sciences,**264*, Article 118502. 10.1016/j.lfs.2020.118502 33031825 10.1016/j.lfs.2020.118502

[53] Riva, F., Bloise, N., Omes, C., Ceccarelli, G., Fassina, L., Nappi, R. E., & Visai, L. (2023). Human ovarian follicular fluid mesenchymal stem cells express osteogenic markers when cultured on bioglass 58S-coated titanium scaffolds. *Materials (Basel),**16*(10), Article 3676. 10.3390/ma16103676 37241304 10.3390/ma16103676 PMC10222050

[54] Moghadam, A. R., Taheri Moghadam, M., Saki, G., & Nikbakht, R. (2020). Utilizing calcium alginate for the assessment of bone morphogenetic protein 15 induction effect on the differentiation of mesenchymal stem cell derived from human follicular fluid to oocyte-like structure. *Advanced Biomedical Research,**9*(1), Article 80. 10.4103/abr.abr_200_20 33912496 10.4103/abr.abr_200_20 PMC8059453

[55] Liao, Z., Zhou, Y., Tao, W., Shen, L., Qian, K., & Zhang, H. (2025). Correlation between the follicular fluid extracellular-vesicle-derived microRNAs and signaling disturbance in the oocytes of women with polycystic ovary syndrome. *Journal Of Ovarian Research,**18*(1), Article 31. 10.1186/s13048-025-01619-0 39966990 10.1186/s13048-025-01619-0 PMC11837631

[56] Devi, S., Bongale, A. M., Tefera, M. A., Dixit, P., & Bhanap, P. (2023). Fresh umbilical cord blood—A source of multipotent stem cells, collection, banking, cryopreservation, and ethical concerns. *Life,**13*(9), Article 1794. 10.3390/life13091794 37763198 10.3390/life13091794 PMC10533013

[57] Haddad, M. E., Karlmark, K. R., Donato, X. C., Martin, G. V., Bretelle, F., Lesavre, N., Cocallemen, J. F., Martin, M., Picard, C., Roudier, J., Desbriere, R., & Lambert, N. C. (2021). Factors predicting the presence of maternal cells in cord blood and associated changes in immune cell composition. *Frontiers in Immunology*, *12*, 651399. 10.3389/fimmu.2021.763236 34659269 10.3389/fimmu.2021.763236 PMC8518619

[58] Mayani, H. (2024). Umbilical cord blood hematopoietic cells: From biology to hematopoietic transplants and cellular therapies. *Archives of Medical Research*, *55*(6), 103042. 10.1016/j.arcmed.2024 39003965 10.1016/j.arcmed.2024.103042

[59] Bonilla, X., Lara, A. M., Llano-León, M., López-González, D. A., Hernández-Mejía, D. G., Bustos, R. H., Camacho-Rodríguez, B., & Perdomo-Arciniegas, A. M. (2023). Mesenchymal stromal cells from perinatal tissues as an alternative for ex vivo expansion of hematopoietic progenitor and stem cells from umbilical cord blood. *International Journal of Molecular Sciences,**24*(21), Article 15544. 10.3390/ijms242115544 37958529 10.3390/ijms242115544 PMC10648510

[60] Shang, Y., Guan, H., & Zhou, F. (2021). Biological characteristics of umbilical cord mesenchymal stem cells and its therapeutic potential for hematological disorders. *Frontiers in Cell and Developmental Biology,**9*, Article 570179. 10.3389/fcell.2021.570179 34012958 10.3389/fcell.2021.570179 PMC8126649

[61] Hordyjewska, A., Popiołek, Ł, & Horecka, A. (2015). Characteristics of hematopoietic stem cells of umbilical cord blood. *Cytotechnology,**67*(3), 387–96. 10.1007/s10616-014-9796-y 25373337 10.1007/s10616-014-9796-y PMC4371573

[62] Sanchez-Petitto, G., Rezvani, K., Daher, M., Rafei, H., Kebriaei, P., Shpall, E. J., & Olson, A. (2023). Umbilical cord blood transplantation: Connecting its origin to its future. *Stem Cells Translational Medicine,**12*(2), 55–71. 10.1093/stcltm/szac086 36779789 10.1093/stcltm/szac086 PMC9985112

[63] Sanz, J., & Rocha, V. (2024). Role of Umbilical Cord Blood Transplantation. In: The EBMT Handbook: Hematopoietic Cell Transplantation and Cellular Therapies 8th edition. Cham (CH) Springer, Sureda A, Corbacioglu S, Greco R, et al., editors. 39437029

[64] Watt, A. P., Kirkland, M., Nekkanti, L., Pham, Y., McDonald, C., Malhotra, A., Moeneclaey, G., Miller, S. L., & Jenkin, G. (2022). Effect of expansion of human umbilical cord blood CD34 + cells on neurotrophic and angiogenic factor expression and function. *Cell and Tissue Research,**388*, 117–132. 10.1007/s00441-022-03592-2 35106623 10.1007/s00441-022-03592-2 PMC8976778

[65] Imahashi, N., Kurita, N., Konuma, T., Takahashi, S., Nishida, T., Tanaka, M., Nakamae, H., Kawakita, T., Ota, S., Doki, N., Onishi, Y., Sawa, M., Ozeki, K., Hiramoto, N., Onizuka, M., Ishimaru, F., Ichinohe, T., Atsuta, Y., & Kanda, J. (2023). Effect of conditioning regimens and Graft-versus-Host disease prophylaxis on the outcomes of umbilical cord blood transplantation performed with Cyclophosphamide/Total body Irradiation-Based regimens. *Transplantation and Cellular Therapy*, *30*(3). 10.1016/j.jtct.2023.12.004 10.1016/j.jtct.2023.12.004 38081416

[66] Wang, J., & Metheny, L. (2023). Umbilical cord blood derived cellular therapy: Advances in clinical development. *Frontiers in Oncology*, *18*, 13:1167266. 10.3389/fonc.2023 10.3389/fonc.2023.1167266 PMC10232824 37274288

[67] Nagamura-Inoue, T., & Nagamura, F. (2023). Umbilical cord blood and cord tissue banking as somatic stem cell resources to support medical cell modalities. *Inflammation and Regeneration,**43*, Article 59. 10.1186/s41232-023-00311-4 38053217 10.1186/s41232-023-00311-4 PMC10696687

[68] Deuse, T., Stubbendorff, M., Tang-Quan, K., Phillips, N., Kay, M. A., Eiermann, T., Phan, T. T., Volk, H. D., Reichenspurner, H., Robbins, R. C., & Schrepfer, S. (2011). Immunogenicity and immunomodulatory properties of umbilical cord lining mesenchymal stem cells. *Cell Transplantation,**20*(5), 655–67. 10.3727/096368910X536473 21054940 10.3727/096368910X536473

[69] Zhang, Y., Pan, Y., Liu, Y., Li, X., Tang, L., Duan, M., Li, J., & Zhang, G. (2021). Exosomes derived from human umbilical cord blood mesenchymal stem cells stimulate regenerative wound healing via transforming growth factor-β receptor Inhibition. *Stem Cell Research & Therapy*, *12*(1), 434. 10.1186/s13287-021-02517-0 34344478 10.1186/s13287-021-02517-0 PMC8336384

[70] Li, X., Zhang, D., Yu, Y., Wang, L., & Zhao, M. (2023). Umbilical cord-derived mesenchymal stem cell secretome promotes skin regeneration and rejuvenation: From mechanism to therapeutics. *Cell Proliferation,**57*(4), Article e13586. 10.1111/cpr.13586 38148579 10.1111/cpr.13586 PMC10984109

[71] Lee, D. H., Kim, S. A., Song, J. S., Shetty, A. A., Kim, B. H., & Kim, S. J. (2022). Cartilage regeneration using human umbilical cord blood derived mesenchymal stem cells: A systematic review and meta-analysis. *Medicina,**58*(12), Article 1801. 10.3390/medicina58121801 36557003 10.3390/medicina58121801 PMC9786930

[72] Dhillon, J., Kraeutler, M. J., Belk, J. W., & Scillia, A. J. (2022). Umbilical cord-derived stem cells for the treatment of knee osteoarthritis: A systematic review. *Orthopaedic Journal of Sports Medicine,**10*(7), Article 23259671221104409. 10.1177/23259671221104409 35859650 10.1177/23259671221104409 PMC9289921

[73] Li, M., Huang, H., Wei, X., Li, H., Li, J., Xie, B., Yang, Y., Fang, X., Wang, L., Zhang, X., Wang, H., Li, M., Lin, Y., Wang, D., Wang, Y., Zhao, T., Sheng, J., Hao, X., Yan, M., … Chang, Z. (2025). Clinical investigation on nebulized human umbilical cord MSC-derived extracellular vesicles for pulmonary fibrosis treatment. *Signal Transduction and Targeted Therapy,**10*, Article 179. 10.1038/s41392-025-02262-3 40461474 10.1038/s41392-025-02262-3 PMC12134356

[74] Lanzoni, G., Linetsky, E., Correa, D., Messinger Cayetano, S., Alvarez, R. A., Kouroupis, D., Alvarez Gil, A., Poggioli, R., Ruiz, P., Marttos, A. C., Hirani, K., Bell, C. A., Kusack, H., Rafkinm, L., Baidal, D., Pastewski, A., Gawri, K., Leñero, C., Mantero, A. M. A., Metalonism, S. W., Wang, X., Roque, L., Masters, B., Kenyon, N. S., Ginzburg, E., Xu, X., Tan, J., Caplan, A. I., Glassberg, M. K., Alejandro, R., & Ricordi, C. (2021). Umbilical cord mesenchymal stem cells for COVID-19 acute respiratory distress syndrome: A double-blind, phase 1/2a, randomized controlled trial. *Stem Cells Translational Medicine*, *10*(5), 660–673. 10.1002/sctm.20-0472 33400390 10.1002/sctm.20-0472 PMC8046040

[75] Gupta, A. O., & Wagner, J. E. (2020). Umbilical cord blood transplants: Current status and evolving therapies. *Frontiers in Pediatrics,**8*, Article 570282. 10.3389/fped.2020.570282 33123504 10.3389/fped.2020.570282 PMC7567024

[76] Ratajczak, M. Z., Zuba-Surma, E. K., Machalinski, B., Ratajczak, J., & Kucia, M. (2008). Very small embryonic-like (VSEL) stem cells: Purification from adult organs, characterization, and biological significance. *Stem Cell Reviews and Reports,**4*(2), 89–99. 10.1007/s12015-008-9018-0 10.1007/s12015-008-9018-0 18459073

[77] Ratajczak, M. Z., Suszynska, M., Pedziwiatr, D., Mierzejewska, K., & Greco, N. J. (2012). Umbilical cord blood-derived very small embryonic like stem cells (VSELs) as a source of pluripotent stem cells for regenerative medicine. *Pediatric Endocrinology Reviews,**9*(3), 639–643. 22523831

[78] Monti, M., Imberti, B., Bianchi, N., Pezzotta, A., Morigi, M., Fante, D., Redi, C., C.A., & Perotti, C. (2017). A novel method for isolation of pluripotent stem cells from human umbilical cord blood. *Stem Cells and Development*, *26*(17), 1258–1269. 10.1089/scd.2017.0012 28583028 10.1089/scd.2017.0012

[79] Bhartiya, D., Jha, N., Tripathi, A., & Tripathi, A. (2023). Very small embryonic-like stem cells have the potential to win the three-front war on tissue damage, cancer, and aging. *Frontiers in Cell and Developmental Biology,**10*, Article 1061022. 10.3389/fcell.2022.1061022 36684436 10.3389/fcell.2022.1061022 PMC9846763

[80] Danova-Alt, R., Heider, A., Egger, D., Cross, M., & Alt, R. (2012). Very small embryonic-like stem cells purified from umbilical cord blood lack stem cell characteristics. *PLoS One,**7*(4), Article e34899. 10.1371/journal.pone.0034899 22509366 10.1371/journal.pone.0034899 PMC3318011

[81] Abbott, A. (2013). Doubt cast over tiny stem cells. *Nature,**499*(7459), 390. 10.1038/499390a 23887410 10.1038/499390a

[82] Miyanishi, M., Mori, Y., Seita, J., Chen, J. Y., Karten, S., Chan, C. K., Nakauchi, H., & Weissman, I. L. (2013). Do pluripotent stem cells exist in adult mice as very small embryonic stem cells? *Stem Cell Reports*, *1*(2), 198–208. 10.1016/j.stemcr.2013.07.001 24052953 10.1016/j.stemcr.2013.07.001 PMC3757755

[83] Kucia, M., Reca, R., Campbell, F. R., Zuba-Surma, E., Majka, M., Ratajczak, J., & Ratajczak, M. Z. (2006). A population of very small embryonic-like (VSEL) CXCR4(+)SSEA-1(+)Oct-4 + stem cells identified in adult bone marrow. *Leukemia,**20*(5), 857–869. 10.1038/sj.leu.2404171 16498386 10.1038/sj.leu.2404171

[84] Zuba-Surma, E. K., & Ratajczak, M. Z. (2010). Overview of very small embryonic-like stem cells (VSELs) and methodology of their identification and isolation by flow cytometric methods. *Current Protocols in Cytometry*, *9*:Unit9.29. 10.1002/0471142956.cy0929s51. Unit9.29. 10.1002/0471142956.cy0929s51 20069527

[85] Thetchinamoorthy, K., Jarczak, J., Kieszek, P., Wierzbicka, D., Ratajczak, J., Kucia, M., & Ratajczak, M. Z. (2025). Very small embryonic-like stem cells (VSELs) on the way for potential applications in regenerative medicine. *Frontiers in Bioengineering and Biotechnology,**13*, Article 1564964. 10.3389/fbioe.2025.1564964 40124247 10.3389/fbioe.2025.1564964 PMC11926153

[86] Shamsnajafabadi, H., & Soheili, Z. S. (2022). Amniotic fluid characteristics and its application in stem cell therapy: A review. *International Journal of Reproductive BioMedicine (IJRM),**20*(8), 627–643. 10.18502/ijrm.v20i8.11752 36313262 10.18502/ijrm.v20i8.11752 PMC9596929

[87] Qiu, C., Ge, Z., Cui, W., Yu, L., & Li, J. (2020). Human amniotic epithelial stem cells: A promising seed cell for clinical applications. *International Journal of Molecular Sciences,**21*(20), Article 7730. 10.3390/ijms21207730 33086620 10.3390/ijms21207730 PMC7594030

[88] Liu, Q. W., Huang, Q. M., Wu, H. Y., Zuo, G. S. L., Gu, H. C., Deng, K. Y., & Xin, H. B. (2021). Characteristics and therapeutic potential of human amnion-derived stem cells. *International Journal of Molecular Sciences,**22*(2), Article 970. 10.3390/ijms22020970 33478081 10.3390/ijms22020970 PMC7835733

[89] Miceli, V., Bertani, A., Chinnici, C. M., Bulati, M., Pampalone, M., Amico, G., Carcione, C., Schmelzer, E., Gerlach, J. C., & Conaldi, P. G. (2021). Conditioned medium from human amnion-derived mesenchymal stromal/stem cells attenuating the effects of cold ischemia-reperfusion injury in an in vitro model using human alveolar epithelial cells. *International Journal of Molecular Sciences,**22*(2), Article 510. 10.3390/ijms22020510 33419219 10.3390/ijms22020510 PMC7825633

[90] Jin, S., Zhang, W., Zeng, W., Zhang, Y., Feng, J., Wang, Y., Luo, H., Liu, T., & Lu, H. (2024). In vitro differentiation of human amniotic epithelial stem cells into keratinocytes regulated by OPN3. *Experimental Dermatology,**33*(1), Article e15007. 10.1111/exd.15007 38284195 10.1111/exd.15007

[91] Maraldi, T., & Russo, V. (2022). Amniotic fluid and placental membranes as sources of stem cells: Progress and challenges. *International Journal of Molecular Sciences,**23*(10), Article 5362. 10.3390/ijms23105362 35628186 10.3390/ijms23105362 PMC9141978

[92] Jiang, F., Zhang, W., Zhou, M., Zhou, Z., Shen, M., Chen, N., & Jiang, X. (2020). Human amniotic mesenchymal stromal cells promote bone regeneration via activating endogenous regeneration. *Theranostics,**10*(14), 6216–6230. 10.7150/thno.45249 32483449 10.7150/thno.45249 PMC7255030

[93] Antounians, L., Tzanetakis, A., Pellerito, O., Catania, V. D., Sulistyo, A., Montalva, L., McVey, M. J., & Zani, A. (2019). The regenerative potential of amniotic fluid stem cell extracellular vesicles: Lessons learned by comparing different isolation techniques. *Scientific Reports,**9*(1), Article 1837. 10.1038/s41598-018-38320-w 30755672 10.1038/s41598-018-38320-w PMC6372651

[94] Chia, W. K., Cheah, F. C., Aziz, A., Kampan, N. H., Shuib, N. C., Khong, S., Tan, T. Y., G.C., & Wong, Y. P. (2021). A review of placenta and umbilical Cord-Derived stem cells and the Immunomodulatory basis of their therapeutic potential in bronchopulmonary dysplasia. *Frontiers in Pediatrics*, *9*, 615508. 10.3389/fped.2021.615508 33791258 10.3389/fped.2021.615508 PMC8006350

[95] Wang, R., Feng, B., Yao, Q., Pan, Q., Yu, J., Liu, C., Wang, J., Li, L., Cao, H., & Xie, J. (2025). Human placenta mesenchymal stromal cells alleviate intestinal inflammation and repair intestinal barrier function by activating AMPK-FXR pathway. *Communications Biology,**8*(1), Article 830. 10.1038/s42003-025-08261-y 40447793 10.1038/s42003-025-08261-y PMC12125195

[96] de Laorden, E. H., Simón, D., Milla, S., Portela-Lomba, M., Mellén, M., Sierra, J., de la Villa, P., Moreno-Flores, M. T., & Iglesias, M. (2023). Human placenta-derived mesenchymal stem cells stimulate neuronal regeneration by promoting axon growth and restoring neuronal activity. *Frontiers in Cell and Developmental Biology*, *11*, 1328261. 10.3389/fcell.2023.1328261 38188022 10.3389/fcell.2023.1328261 PMC10766706

[97] Biswas, A., Rajasekaran, R., Saha, B., Dixit, K., Vaidya, P. V., Ojha, A. K., & Dhara, S. (2023). Human placenta/umbilical cord derivatives in regenerative medicine - Prospects and challenges. *Biomaterials Science,**11*(14), 4789–4821. 10.1039/d2bm01977a 37255413 10.1039/d2bm01977a

[98] Fang, Y. H., Wang, S. P. H., Chang, H. Y., Yang, P. J., Liu, P. Y., & Liu, Y. W. (2020). Progress and challenges of amniotic fluid derived stem cells in therapy of ischemic heart disease. *International Journal of Molecular Sciences,**22*(1), Article 102. 10.3390/ijms22010102 33374215 10.3390/ijms22010102 PMC7794729

[99] Edström, D., Niroomand, A., Stenlo, M., Broberg, E., Hirdman, G., Ghaidan, H., Hyllén, S., Pierre, L., Olm, F., & Lindstedt, S. (2024). Amniotic fluid-derived mesenchymal stem cells reduce inflammation and improve lung function following transplantation in a porcine model. *The Journal of Heart and Lung Transplantation,**43*(12), 2018–2030. 10.1016/j.healun.2024.08.014 39182800 10.1016/j.healun.2024.08.014

[100] Gatti, M., Beretti, F., Zavatti, M., Bertucci, E., Ribeiro Luz, S., Palumbo, C., & Maraldi, T. (2020). Amniotic fluid stem cell-derived extracellular vesicles counteract steroid-induced osteoporosis in vitro. *International Journal of Molecular Sciences,**22*(1), Article 38. 10.3390/ijms22010038 33375177 10.3390/ijms22010038 PMC7792960

[101] Stefańska, K., Ożegowska, K., Hutchings, G., Popis, M., Moncrieff, L., Dompe, C., Janowicz, K., Pieńkowski, W., Gutaj, P., Shibli, J. A., Prado, W. M., Piotrowska-Kempisty, H., Mozdziak, P., Bruska, M., Zabel, M., Kempisty, B., & Nowicki, M. (2020). Human wharton’s jelly-cellular specificity, stemness potency, animal models, and current application in human clinical trials. *Journal of Clinical Medicine,**9*(4), Article 1102. 10.3390/jcm9041102 32290584 10.3390/jcm9041102 PMC7230974

[102] Marino, L., Castaldi, M. A., Rosamilio, R., Ragni, E., Vitolo, R., Fulgione, C., Castaldi, S. G., Serio, B., Bianco, R., Guida, M., & Selleri, C. (2019). Mesenchymal stem cells from the wharton’s jelly of the human umbilical cord: Biological properties and therapeutic potential. *International Journal of Stem Cells,**12*(2), 218–226. 10.15283/ijsc18034 31022994 10.15283/ijsc18034 PMC6657936

[103] Stefańska, K., Nemcova, L., Blatkiewicz, M., Pieńkowski, W., Ruciński, M., Zabel, M., Mozdziak, P., Podhorska-Okołów, M., Dzięgiel, P., & Kempisty, B. (2023). Apoptosis related human wharton’s jelly-derived stem cells differentiation into osteoblasts, chondrocytes, adipocytes and neural-like cells—Complete transcriptomic assays. *International Journal of Molecular Sciences,**24*(12), Article 10023. 10.3390/ijms241210023 37373173 10.3390/ijms241210023 PMC10297881

[104] Drobiova, H., Sindhu, S., Ahmad, R., Haddad, D., Al-Mulla, F., & Madhoun, A., A (2023). Wharton’s jelly mesenchymal stem cells: A concise review of their secretome and prospective clinical applications. *Frontiers in Cell and Developmental Biology*, *11*, 1211217. 10.3389/fcell.2023.1211217 37440921 10.3389/fcell.2023.1211217 PMC10333601

[105] Niknam, B., Baghaei, K., Mahmoud Hashemi, S., Hatami, B., Reza Zali, M., & Amani, D. (2023). Human Wharton’s jelly mesenchymal stem cells derived-exosomes enriched by miR-124 promote an anti-fibrotic response in an experimental model of liver fibrosis. *International Immunopharmacology*, 119,110294. 10.1016/j.intimp.2023 10.1016/j.intimp.2023.110294 37167639

[106] Patel, A. A., Mohamed, A. H., Rizaev, J., Mallick, A. K., Qasim, M. T., Abdulmonem, W. A., Jamal, A., Hattiwale, H. M., Kamal, M. A., & Ahmad, F. (2024). Application of mesenchymal stem cells derived from the umbilical cord or wharton’s jelly and their extracellular vesicles in the treatment of various diseases. *Tissue and Cell*, *89*, 102415. 10.1016/j.tice.2024 38851032 10.1016/j.tice.2024.102415

[107] Millán-Rivero, J. E., Nadal-Nicolás, F. M., García-Bernal, D., Sobrado-Calvo, P., Blanquer, M., Moraleda, J. M., Vidal-Sanz, M., & Agudo-Barriuso, M. (2018). Human wharton’s jelly mesenchymal stem cells protect axotomized rat retinal ganglion cells via secretion of anti-inflammatory and neurotrophic factors. *Scientific Reports,**8*(1), Article 16299. 10.1038/s41598-018-34527-z 30389962 10.1038/s41598-018-34527-z PMC6214908

[108] Chang, Y. H., Vuong, C. K., Ngo, N. H., Yamashita, T., Ye, X., Futamura, Y., Fukushige, M., Obata-Yasuoka, M., Hamada, H., Osaka, M., Hiramatsu, Y., Sakurai, T., & Ohneda, O. (2022). Extracellular vesicles derived from Wharton’s jelly mesenchymal stem cells inhibit the tumor environment via the miR-125b/HIF1α signaling pathway. *Scientific Reports,**12*(1), Article 13550. 10.1038/s41598-022-17767-y 35941273 10.1038/s41598-022-17767-y PMC9359975

[109] Wyatt, K. A., Filby, C. E., Davies-Tuck, M. L., Suke, S. G., Evans, J., & Gargett, C. E. (2021). Menstrual fluid endometrial stem/progenitor cell and supernatant protein content: Cyclical variation and indicative range. *Human Reproduction,**36*(8), 2215–2229. 10.1093/humrep/deab156 34173001 10.1093/humrep/deab156

[110] Tindal, K., Filby, C. E., Cousins, F. L., Ellery, S. J., Vollenhoven, B., Palmer, K., Gordon, A., Gargett, C. E., & Davies-Tuck, M. (2024). The composition of menstrual fluid, its applications, and recent advances to understand the endometrial environment: A narrative review. *F&S Reviews,**5*(3), Article 100075. 10.1016/j.xfnr.2024.100075

[111] Domnina, A., Novikova, P., Obidina, J., Fridlyanskaya, I., Alekseenko, L., Kozhukharova, I., Lyublinskaya, O., Zenin, V., & Nikolsky, N. (2018). Human mesenchymal stem cells in spheroids improve fertility in model animals with damaged endometrium. *Stem Cell Research & Therapy*, 9, 50. 10.1186/s13287-018-0801-9 10.1186/s13287-018-0801-9 PMC5828181 29482664

[112] Liu, Y., Niu, R., Yang, F., Yan, Y., Liang, S., Sun, Y., Shen, P., & Lin, J. (2018). Biological characteristics of human menstrual blood-derived endometrial stem cells. *Journal of Cellular and Molecular Medicine,**22*, 1627–1639. 10.1111/jcmm.13437 29278305 10.1111/jcmm.13437 PMC5824373

[113] Gargett, C. E., Schwab, K. E., & Deane, J. A. (2016). Endometrial stem/progenitor cells: The first 10 years. *Human Reproduction Update*, *22*(2), 137-63. 10.1093/humupd/dmv051. 10.1093/humupd/dmv051 PMC4755439 26552890

[114] Chen, L., Qu, J., & Xiang, C. (2019). The multi-functional roles of menstrual blood-derived stem cells in regenerative medicine. *Stem Cell Research & Therapy*, *10*, 1. 10.1186/s13287-018-1105-9 30606242 10.1186/s13287-018-1105-9 PMC6318883

[115] He, Y., Han, Y., & Ye, Y. (2022). Therapeutic potential of menstrual blood-derived stem cell transplantation for intrauterine adhesions. *Frontiers in Surgery,**9*, Article 847213. 10.3389/fsurg.2022.847213 35274000 10.3389/fsurg.2022.847213 PMC8901573

[116] Sheikholeslami, A., Kalhor, N., Sheykhhasan, M., Jannatifar, R., & Sahraei, S. S. (2021). Evaluating differentiation potential of the human menstrual blood-derived stem cells from infertile women into oocyte-like cells. *Reproductive Biology,**21*, Article 100477. 10.1016/j.repbio.2020.100477 33401233 10.1016/j.repbio.2020.100477

[117] Zafardoust, S., Kazemnejad, S., Darzi, M., Fathi-Kazerooni, M., Saffarian, Z., Khalili, N., Edalatkhah, H., Mirzadegan, E., & Khorasani, S. (2023). Intraovarian administration of autologous menstrual blood derived-mesenchymal stromal cells in women with premature ovarian failure. *Archives Of Medical Research,**54*(2), 135–144. 10.1016/j.arcmed.2022.12.015 36702667 10.1016/j.arcmed.2022.12.015

[118] Fang, Y., Chen, L., Yuan, Y., Zhou, S., Fu, J., Zhang, Q., Zhang, N., Huang, Y., Li, Y., Yuan, L., Chen, L., & Xiang, C. (2025). Human menstrual blood-derived stem cells secreted ECM1 directly interacts with LRP1α to ameliorate hepatic fibrosis through FoxO1 and mTOR signaling pathway. *Stem Cell Research & Therapy*, *16*(1), 230. 10.1186/s13287-025-04351-0 40336034 10.1186/s13287-025-04351-0 PMC12060366

[119] Liu, Y., Niu, R., Li, W., Lin, J., Stamm, C., Steinhoff, G., & Ma, N. (2019). Therapeutic potential of menstrual blood-derived endometrial stem cells in cardiac diseases. *Cellular and Molecular Life Sciences,**76*(9), 1681–1695. 10.1007/s00018-019-03019-2 30721319 10.1007/s00018-019-03019-2 PMC11105669

[120] Ruan, T., Han, J., Xue, C., Wang, F., & Lin, J. (2025). Mesenchymal stem cells protect the integrity of the alveolar epithelial barrier through extracellular vesicles by inhibiting MAPK-mediated necroptosis. *Stem Cell Research & Therapy*, *16*, 250. 10.1186/s13287-025-04388-1 40390004 10.1186/s13287-025-04388-1 PMC12090679

[121] Yu, L., Wu, H., Zeng, S., Hu, X., Wu, Y., Zhou, J., Yuan, L., Zhang, Q., Xiang, C., & Feng, Z. (2024). Menstrual blood-derived mesenchymal stem cells combined with collagen I gel as a regenerative therapeutic strategy for degenerated disc after discectomy in rats. *Stem Cell Research & Therapy*, *15*(1), 75. 10.1186/s13287-024-03680-w 38475906 10.1186/s13287-024-03680-w PMC10935903

[122] Robalo Cordeiro, M., Roque, R., Laranjeiro, B., Carvalhos, C., & Figueiredo-Dias, M. (2024). Menstrual blood stem cells-derived exosomes as promising therapeutic tools in premature ovarian insufficiency induced by gonadotoxic systemic anticancer treatment. *International Journal of Molecular Sciences,**25*(15), Article 8468. 10.3390/ijms25158468 39126037 10.3390/ijms25158468 PMC11312895

[123] Chen, L., Xiang, B., Wang, X., & Xiang, C. (2017). Exosomes derived from human menstrual blood-derived stem cells alleviate fulminant hepatic failure. *Stem Cell Research & Therapy*. 10.1186/s13287-016-0453-6. 8,9. 10.1186/s13287-016-0453-6 PMC5260032 28115012

[124] Davoodi Asl, F., Sahraei, S. S., Kalhor, N., Fazaeli, H., Sheykhhasan, M., Soleimani Moud, S., Naserpour, L., & Sheikholeslami, A. (2023). Promising effects of exosomes from menstrual blood-derived mesenchymal stem cells on endometriosis. *Reproductive Biology*, *23*(3), 100788. 10.1016/j.repbio.2023 37542905 10.1016/j.repbio.2023.100788

[125] Yildirim, S. (2024). Dental pulp is a connective tissue. *Dental pulp derived mesenchymal stromal cells*. Springer. 10.1007/978-1-0716-4244-3_4

[126] Ghannam, M. G., Alameddine, H., & Bordoni, B. (2025). Anatomy, Head and Neck, Pulp (Tooth). StatPearls Publishing. https://www.ncbi.nlm.nih.gov/books/NBK537112 30725797

[127] Pohl, S., Akamp, T., Smeda, M., Uderhardt, S., Besold, D., Krastl, G., Galler, K. M., Buchalla, W., & Widbiller, M. (2024). Understanding dental pulp inflammation: From signaling to structure. *Frontiers in Immunology*, *15*, 1474466. 10.3389/fimmu.2024 39534600 10.3389/fimmu.2024.1474466 PMC11554472

[128] Monti, M., Graziano, A., Rizzo, S., Perotti, C., Del Fante, C., d’Aquino, R., Redi, C. A., Rodriguez, Y., & Baena, R. (2016). In vitro and in vivo differentiation of progenitor stem cells obtained after mechanical digestion of human dental pulp. *Journal of Cellular Physiology*, *232*(3), 548–555. 10.1002/jcp.25452 27277190 10.1002/jcp.25452

[129] Kok, Z. Y., Alaidaroos, N. Y. A., Alraies, A., Colombo, J. S., Davies, L. C., Waddington, R. J., Sloan, A. J., & Moseley, R. (2022). Dental pulp stem cell heterogeneity: Finding superior quality needles in a dental pulpal haystack for regenerative medicine-based applications. *Stem Cells International,**2022*, Article 9127074. 10.1155/2022/9127074 35027930 10.1155/2022/9127074 PMC8752304

[130] Yu, J., Wang, Y., Deng, Z., Tang, L., Li, Y., Shi, J., & Jin, Y. (2007). Odontogenic capability: Bone marrow stromal stem cells versus dental pulp stem cells. *Biology of the Cell,**99*(8), 465–474. 10.1042/BC20070013 17371295 10.1042/BC20070013

[131] Stefańska, K., Volponi, A. A., Kulus, M., Waśko, J., Farzaneh, M., Grzelak, J., Azizidoost, S., Mozdziak, P., Bukowska, D., Antosik, P., Zabel, M., Podhorska-Okołów, M., Dzięgiel, P., Szcześniak, M., Woszczyk, M., & Kempisty, B. (2024). Dental pulp stem cells - A basic research and future application in regenerative medicine. *Biomedicine & Pharmacotheraphy*. 10.1016/j.biopha.2024.116990. 178,116990. 10.1016/j.biopha.2024.116990 39024839

[132] Monteiro, N., & Yelick, P. C. (2017). Advances and perspectives in tooth tissue engineering. *Journal of Tissue Engineering and Regenerative Medicine*, *11*(9), 2443–2461. 10.1002/term.2134 27151766 10.1002/term.2134 PMC6625321

[133] Sequeira, D. B., Diogo, P., Gomes, B. P. F. A., Peça, J., & Santos, J. M. M. (2024). Scaffolds for Dentin–Pulp complex regeneration. *Medicina*, *60*(1), 7. 10.3390/medicina60010007 10.3390/medicina60010007 PMC10821321 38276040

[134] Tsutsui, T. W. (2020). Dental pulp stem cells: Advances to applications. *Stem Cells Cloning,**13*, 33–42. 10.2147/SCCAA.S166759 32104005 10.2147/SCCAA.S166759 PMC7025818

[135] Spagnuolo, G., De Luca, I., Iaculli, F., Barbato, E., Valletta, A., Calarco, A., Valentino, A., & Riccitiello, F. (2023). Regeneration of dentin-pulp complex: Effect of calcium-based materials on hDPSCs differentiation and gene expression. *Dental Materials,**39*(5), 485–491. 10.1016/j.dental.2023.03.017 36935304 10.1016/j.dental.2023.03.017

[136] Zhao, J., Zhou, Y. H., Zhao, Y. Q., Gao, Z. R., Ouyang, Z. Y., Ye, Q., Liu, Q., Chen, Y., Tan, L., Zhang, S. H., Feng, Y., Hu, J., Dusenge, M. A., Feng, Y. Z., & Guo, Y. (2023). Oral cavity-derived stem cells and preclinical models of jaw-bone defects for bone tissue engineering. *Stem Cell Research & Therapy*. 10.1186/s13287-023-03265-z. 14,39. 10.1186/s13287-023-03265-z PMC10022059 36927449

[137] Wang, K., Liu, X., Jiang, X., Chen, S., Wang, H., Wang, Z., Wang, Q., & Li, Z. (2025). Human dental pulp stem cells for spinal cord injury. *Stem Cell Research & Therapy*, 16123. 10.1186/s13287-025-04244-2 10.1186/s13287-025-04244-2 PMC11887269 40055766

[138] Wen, X., Jiang, W., Li, X., Liu, Q., Kang, Y., & Song, B. (2024). Advancements in spinal cord injury repair: Insights from dental-derived stem cells. *Biomedicines,**12*(3), Article 683. 10.3390/biomedicines12030683 38540295 10.3390/biomedicines12030683 PMC10968527

[139] Anderson, S., Prateeksha, P., & Das, H. (2022). Dental pulp-derived stem cells reduce inflammation, accelerate wound healing and mediate M2 polarization of myeloid cells. *Biomedicines*. 10.3390/biomedicines10081999 36009546 10.3390/biomedicines10081999 PMC9624276

[140] Fageeh, H. N. (2021). Preliminary evaluation of proliferation, wound healing properties, osteogenic and chondrogenic potential of dental pulp stem cells obtained from healthy and periodontitis affected teeth. *Cells,**10*(8), Article 2118. 10.3390/cells10082118 34440887 10.3390/cells10082118 PMC8393753

[141] Hirose, Y., Yamamoto, T., Nakashima, M., Funahashi, Y., Matsukawa, Y., Yamaguchi, M., Kawabata, S., & Gotoh, M. (2016). Injection of dental pulp stem cells promotes healing of damaged bladder tissue in a rat model of chemically induced cystitis. *Cell Transplantation,**25*(3), 425–436. 10.3727/096368915X689523 26395427 10.3727/096368915X689523

[142] Song, B., Jiang, W., Alraies, A., Liu, Q., Gudla, V., Oni, J., Wei, X., Sloan, A., Ni, L., & Agarwal, M. (2016). Bladder smooth muscle cells differentiation from dental pulp stem cells: Future potential for bladder tissue engineering. *Stem Cells International,**2016*, Article 6979368. 10.1155/2016/6979368 26880982 10.1155/2016/6979368 PMC4736571

[143] Min, Q., Yang, L., Tian, H., Tang, L., Xiao, Z., & Shen, J. (2023). Immunomodulatory mechanism and potential application of dental pulp-derived stem cells in immune-mediated diseases. *International Journal of Molecular Sciences,**24*(9), Article 8068. 10.3390/ijms24098068 37175774 10.3390/ijms24098068 PMC10178746

[144] Agrawal, S., Vaidya, S., Patel, J., Jirvankar, P., & Gurjar, P. (2025). Challenges and pathways in regulating Next-Gen biological therapies. *Current Pharmaceutical Biotechnology*, *23*. 10.2174/0113892010367028250411111549 10.2174/0113892010367028250411111549 40277057

[145] Dahiya, A., Singh, K., Ashish, A., Nipun, Bhadyaria, A., Thakur, S., Kumar, M., Das Gupta, G., Kurmi, D., B., & Pal, R. R. (2025). Global harmonization in advanced therapeutics: Balancing innovation, safety, and access. *Personalized Medicine*, *22*(3), 181–191. 10.1080/17410541.2025.2494980 40277251 10.1080/17410541.2025.2494980

[146] Kakroodi, F. A., Khodadoust, E., Alizadeh, M., Hayaei Tehrani, R. S., Sarabi, P. A., Rahmanian, M., & Vosough, M. (2025). Current challenges and future directions of ATMPs in regenerative medicine. *Regenerative Therapy,**9*, Article 30:358–370. 10.1016/j.reth.2025.06.017 10.1016/j.reth.2025.06.017 PMC12274769 40689373

