# Supplementary material for: From Biological Waste to Therapeutic Resources: A Comprehensive Review of Stem Cell Sources, Characterization, and Biomedical Potentials
Source: Stem Cell Rev Rep. 2025 Oct 15;22(1):5–25. doi: 10.1007/s12015-025-10989-3 (PMC12795948; doi:10.1007/s12015-025-10989-3)
Supplement: Supplementary file 1 — Supplementary Material 1 (DOCX 26.4 KB) [file 12015_2025_10989_MOESM1_ESM.docx]

**Supplementary table 1**: Ongoing and completed clinical trials investigating the therapeutic potential of stem cells isolated from biological waste materials (urine, USCs, adipose tissue, ADSCs, follicular fluid, FF, cord blood, UCB, placenta PMSCs, amniotic fluid, AFSCs, Wharton’s jelly, WJ-MSCs, menstrual blood, MenSCs , and dental pulp, DPSc. Cell source, reporting registry number, title, brief description, clinical phase, recruitment status, and country categorize trials. “Unknown status” indicates studies with registries not updated for more than two years.

| **Source** | **NCT / Registry** | **Title** | **Brief Summary** | **Phase** | **Status** | **Country** |
| --- | --- | --- | --- | --- | --- | --- |
| USCs | NCT06071143 | Safety and Efficacy of KDSTEM Inj. in Patients With Chronic Kidney Disease | Chronic kidney disease – evaluation of safety, tolerability, and preliminary efficacy of autologous urine-derived stem cells (KDSTEM Inj.) in a Phase 1 dose-escalation study | I | Recruiting | South Korea |
| ADSCs | NCT04280003 | Allogeneic Adipose Tissue-derived Mesenchymal Stem Cells in Ischemic Stroke (AMASCIS-02) | Acute ischemic stroke – evaluation of safety and efficacy of intravenous allogeneic adipose tissue-derived mesenchymal stem cells administered within 4 days of stroke onset | II | Unknown status | Spain |
| ADSCs | NCT03869229 | Adipose-derived Mesenchymal Stem Cells in Osteoarthritis | Mild to moderate osteoarthritis of the hip, knee, or glenohumeral joint – evaluation of safety and efficacy of repeated intra-articular injections of autologous adipose-derived mesenchymal stem cells (AD-MSCs) | I/II | Unknown status | Poland |
| ADSCs | NCT04208646 | Allogenic Adipose Tissue-Derived Mesenchymal Progenitor Cells for the Treatment of Knee Osteoarthritis | Knee osteoarthritis with cartilage defects – evaluation of safety and efficacy of AlloJoin® (allogeneic adipose tissue-derived mesenchymal progenitor cells) therapy | II | Completed | China |
| ADSCs | NCT03956719 | Treatment of Early Knee Osteoarthritis With Autologous Adipose-derived Mesenchymal Stem Cells | Early knee osteoarthritis – evaluation of safety and efficacy of intra-articular autologous adipose-derived mesenchymal stem cell (AD-MSC) injections for pain relief, functional improvement, and cartilage regeneration | Not applicable | Unknown status | China |
| ADSCs | NCT04212728 | Treatment of Knee Osteoarthritis With Autologous Adipose-derived Mesenchymal Stem Cells | Severe knee osteoarthritis – evaluation of safety and efficacy of intra-articular injections of autologous adipose-derived mesenchymal stem cells (AMSCs) combined with platelet-rich plasma (PRP) | Not applicable | Unknown status | China |
| ADSCs | NCT06570291 | Allogenic Adipose-Derived Mesenchymal Stem Cells for the Treatment of Knee Osteoarthritis | Knee osteoarthritis – evaluation of efficacy and safety of allogeneic adipose tissue-derived mesenchymal stem cell (AD-MSC) therapy | III | Recruiting | China |
| ADSCs | NCT04448106 | Autologous Adipose Tissue-Derived Mesenchymal Stem Cells (AdMSCs) for Osteoarthritis (AdMSCs) | Osteoarthritis of the knee, hip, and shoulder – Phase II open-label RCT evaluating safety and efficacy of adipose-derived mesenchymal stem cell therapy across multiple joints | II | Not yet recruiting | USA |
| ADSCs | NCT03608579 | Autologous Culture Expanded Adipose Derived MSCs for Treatment of Painful Hip OA | Evaluation of autologous adipose-derived MSC injections for mild to moderate hip osteoarthritis, assessing safety, efficacy, and comparison of single versus repeated dosing regimens | I | Active, not recruiting | USA |
| ADSCs | NCT03955497 | Effectiveness of Autologous Adipose-derived Stem Cells in the Treatment of Knee Cartilage Injury | Evaluation of autologous adipose-derived MSC gel combined with high tibial osteotomy for cartilage repair in knee osteoarthritis, aiming to improve functional recovery and joint regeneration compared with standard treatment | I/II | Unknown status | China |
| ADSCs | NCT04744051 | ATCell™ Expanded Autologous, Adipose-Derived Mesenchymal Stem Cells Deployed Via Intravenous Infusion | Post-Concussion Syndrome (PCS) / Chronic Concussive Syndrome (CCS) – Phase 1 evaluation of safety, tolerability, and preliminary efficacy of autologous adipose-derived stem cell therapy (ATCell™) | I | Unknown status | USA |
| ADSCs | NCT03913572 | Treatment of Perianal Disease Using Adipose-derived Stem Cells | Chronic perianal disease (including perianal fistulas) – evaluation of safety and efficacy of adipose-derived stem cells (ASCs) as adjunct to surgical treatment compared with traditional techniques | Observational | Unknown status | USA |
| ADSCs | NCT03308565 | Adipose Stem Cells for Traumatic Spinal Cord Injury (CELLTOP) | Severe traumatic spinal cord injury (SCI) – evaluation of safety and feasibility of intrathecal administration of autologous adipose-derived mesenchymal stem cells (AD-MSCs) | I | Completed | USA |
| ADSCs | NCT04520373 | Autologous Adipose Derived Mesenchymal Stem Cells for Spinal Cord Injury Patients | Traumatic spinal cord injury with paralysis – Phase II evaluation of safety and therapeutic potential of intrathecal autologous adipose-derived mesenchymal stem cell (AD-MSC) injections | II | Active, not recruiting | USA |
| ADSCs | NCT04170426 | Autologous Adipose-derived Stem Cells (AdMSCs) for Rheumatoid Arthritis | Rheumatoid arthritis – evaluation of safety and efficacy of intravenous autologous adipose-derived mesenchymal stem cells (AdMSCs, Celltex) in a combined Phase 1/2a trial | I/II | Not yet recruiting | USA |
| ADSCs | NCT03268603 | Intrathecal Autologous Adipose-derived Mesenchymal Stromal Cells for Amyotrophic Lateral Sclerosis (ALS) | Evaluation of intrathecal autologous adipose-derived MSCs for amyotrophic lateral sclerosis (ALS), aiming to assess safety, tolerability, and potential neuroprotective effects through repeated administration to cerebrospinal fluid | II | Active, not recruiting | USA |
| ADSCs | NCT03321942 | Treatment of Chronic Renal Failure With Adipose Tissue-derived Mesenchymal Stem Cells | Chronic renal failure – evaluation of safety and therapeutic effects of adipose-derived mesenchymal stem cells (AMSCs) on oxidative stress, inflammation, mitochondrial damage, and renal function | Not applicable | Unknown status | China |
| ADSCs | NCT04392206 | AMSC for Reducing Anastomotic Stenosis in Primary Arteriovenous Anastomoses | Hemodialysis vascular access – evaluation of safety and efficacy of allogeneic adipose-derived mesenchymal stem cells (AD-MSCs) for arteriovenous fistula and arterial bypass creation, with focus on access maturation and primary anastomotic patency | I | Recruiting | USA |
| ADSCs | NCT03279796 | Treatment of Tendon Disease Using Autologous Adipose-derived Mesenchymal Stem Cells | Rotator cuff injury and lateral epicondylosis (tennis elbow) – evaluation of safety and efficacy of adipose-derived mesenchymal stem cell injections versus betamethasone | II | Unknown status | China |
| ADSCs | NCT05279157 | Autologous Adipose-Derived Adult Stem Cell Implantation for Corneal Diseases (ADASCs-CT-CD) (ADASCs-CT-CD) | Corneal diseases (keratoconus, corneal dystrophies) treated with autologous ADASCs ± decellularized stromal scaffolds | II | Completed | Spain |
| ADSCs | NCT03265613 | Safety and Efficacy of Expanded Allogeneic AD-MSCs in Patients With Moderate to Severe Psoriasis (ADMSP) | Assessment of autologous adipose-derived MSCs for moderate to severe psoriasis, focusing on safety, immunomodulatory effects, and clinical improvement in PASI score, relapse rate, and quality of life | I/II | Completed | China |
| ADSCs | NCT03865394 | Treatment of Chronic Wounds in Diabetic Foot Syndrome With Allogeneic Adipose Derived Mesenchymal Stem Cells (1ABC) | Evaluation of allogeneic adipose-derived MSCs suspended in fibrin solution for the treatment of chronic wounds in diabetic foot syndrome, aiming to promote wound healing and improve clinical outcomes | I/II | Completed | Poland |
| FF-MSCs | NCT01649752 | Role of Stem Cells in Improving Implantation Rates in ICSI Patients | Recurrent implantation failure / poor endometrial receptivity in ICSI patients | I | Unknown status | Egypt |
| FF-MSCs | NCT04382872 | Follicular Fluid Exosome miRNA During Oocyte Maturation | Oocyte maturation physiology | Observational | Recruiting | China |
| UCB-MSCs | NCT01549665 | Umbilical Cord Blood-derived Mesenchymal Stem Cells for the Treatment of Steroid-refractory Acute or Chronic Graft-versus-host-disease (GVHD-MSC) | Steroid-refractory acute or chronic graft-versus-host disease (GVHD) in pediatric patients following allo-HSCT | I/II | Unknown status | South Korea |
| UCB-MSCs | NCT05308342 | Clinical Study of Human Umbilical Cord Mesenchymal Stem Cells in the Treatment of Premature Ovarian Insufficiency | Primary ovarian insufficiency – evaluation of umbilical cord-derived MSC transplantation combined with hormone replacement therapy | Not applicable | Unknown status | China |
| UCB-MSCs | NCT04811651 | Umbilical Cord-derived Mesenchymal Stem Cells for Ischemic Stroke (UMSIS) | Subacute ischemic stroke – evaluation of intravenous UC-MSC infusion | II | Completed | China |
| UCB-MSCs | NCT06518902 | Umbilical Cord-derived Mesenchymal Stem Cell Infusion for Treating Acute Ischemic Stroke (UMERIS) | Acute ischemic stroke – evaluation of safety, efficacy, and dose-limiting toxicity of intravenous umbilical cord-derived MSC injections | I | Not yet recruiting | China |
| UCB-MSCs | NCT05682586 | UC-MSCs in the Treatment of Severe and Critical COVID-19 Patients | Assessment of the safety, efficacy, and immune-modulatory mechanisms of umbilical cord mesenchymal stem cell (UC-MSC) infusions in patients with severe and critical COVID-19 | III | Unknown status | China |
| UCB-MSCs | NCT04565665 | Study of Cord Blood Derived Mesenchymal Stem Cells for Treatment of Moderate, Severe or Critical Pneumonia | Evaluation of the safety and efficacy of cord blood-derived mesenchymal stem cell (CB-MSC) infusions in patients with COVID-19-related acute respiratory distress syndrome (ARDS), aiming to reduce inflammation, and improve oxygenation | I/II | Recruiting | USA |
| UCB-MSCs | NCT06788470 | Safety and Efficacy of Umbilical Cord-derived Mesenchymal Stem Cell(MSC) Transplantation in the Treatment of Bronchopulmonary Dysplasia(BPD) in Premature Infants (MSC，BPD) | Severe bronchopulmonary dysplasia (BPD) in very low and extremely preterm infants, with the goal of improving survival, promoting alveolar and pulmonary vascular development, reducing fibrosis, and enhancing long-term respiratory and neurodevelopmental outcomes | I/II | Recruiting | China |
| UCB-MSCs | NCT03601416 | Intravenous Human Umbilical-Cord-Derived Mesenchymal Stem Cells For Moderate and Severe Bronchopulmonary Dysplasias in Children | Moderate to severe bronchopulmonary dysplasia (BPD) in preterm infants | II | Unknown status | China |
| UCB-MSCs | NCT03608592 | Human Umbilical Cord Mesenchymal Stem Cells (MSCs) Therapy in ARDS (ARDS) | Adult moderate/severe ARDS – evaluation of intravenous UC-MSC infusion as salvage therapy | Not applicable | Unknown status | China |
| UCB-MSCs | NCT04494386 | Umbilical Cord Lining Stem Cells (ULSC) in Patients With COVID-19 ARDS (ULSC) | COVID-19-related acute respiratory distress syndrome (ARDS) | I/II | Completed | USA |
| UCB-MSCs | NCT04014166 | Study of Human Umbilical Cord-derived Mesenchymal Stem Cells for Treatment of Refractory Immune Thrombocytopenia | Refractory immune thrombocytopenia – evaluation of intravenous hUC-MSC infusion | Not applicable | Completed | China |
| UCB-MSCs | NCT03219801 | Umbilical Cord Derived Mesenchymal Stem Cells Therapy in Systemic Lupus Erythematosus | Systemic lupus erythematosus – evaluation of intravenous allogeneic hUC-MSC infusion | Early I | Unknown status | China |
| UCB-MSCs | NCT04356287 | Treatment With Human Umbilical Cord-derived Mesenchymal Stromal Cells in Systemic Sclerosis (CARE-SSc) | Systemic sclerosis – evaluation of safety and efficacy of intravenous UC-MSC infusions | I/II | Recruiting | Canada |
| UCB-MSCs | NCT03055078 | Umbilical Cord Derived Mesenchymal Stem Cells Therapy in Aplastic Anemia | Immune-mediated aplastic anemia – evaluation of intravenous allogeneic UC-MSC infusion | I | Unknown status | China |
| UCB-MSCs | NCT03550183 | Umbilical Cord Derived Mesenchymal Stem Cells Therapy in Parkinson's Disease | Parkinson’s disease (neurodegenerative movement disorder) | I | Unknown status | China |
| UCB-MSCs | NCT05152290 | Safety of Cultured Allogeneic Adult Umbilical Cord Derived Mesenchymal Stem Cells for SCI | Spinal cord injury (SCI) – evaluation of intravenous and intrathecal UC-MSC administration | I | Recruiting | Greece |
| UCB-MSCs | NCT05984303 | Human Umbilical Cord-derived Mesenchymal Stem Cells for Decompensated Cirrhosis (MSC-DLC-1b) | Decompensated cirrhosis – evaluation of intravenous hUC-MSC infusion | I | Not yet recruiting | China |
| UCB-MSCs | NCT05794425 | Clinical Study of UCB Combined With UC-MSCs in the Treatment of Bone Marrow Failure Disorders | Bone marrow failure syndromes | I/II | Recruiting | China |
| UCB-HSCs | NCT03173937 | Unrelated Umbilical Cord Blood Transplantation for Severe Aplastic Anemia and Hypo-plastic MDS Using CordIn(TM), Umbilical Cord Blood-Derived Ex Vivo Expanded Stem and Progenitor Cells to Expedite Engraftment and Improve Transplant Outcome | Severe aplastic anemia (SAA) and myelodysplastic syndrome (MDS) – evaluation of CordIn™ expanded UCB transplantation | I/II | Recruiting | USA |
| UCB-HSCs | NCT01711788 | Intrabone Infusion of Umbilical Cord Blood Stem Cells | Assessment of intrabone infusion of umbilical cord blood hematopoietic stem cells to enhance and accelerate hematopoietic reconstitution after UCB transplantation in pediatric patients with malignant and non-malignant hematologic, immunodeficiency, or metabolic diseases | II | Completed | Canada |
| UCB-HSCs | NCT03004976 | Study of Allogeneic Umbilical Cord Blood Infusion for Adults With Ischemic Stroke (CoBIS 2) | Evaluation of a single intravenous infusion of unrelated donor umbilical cord blood to improve functional recovery, neurological outcomes, quality of life, and cognitive status in adult patients with recent ischemic stroke (3–10 days post-event) | II | Completed | USA |
| hAESCs | NCT05691114 | Precise Transplantation of Human Amniotic Epithelial Stem Cells Into Lateral Ventricle for Parkinson's Disease | Idiopathic Parkinson’s disease – evaluation of intraventricular hAESC infusion (dose-escalation study) | I | Recruiting | China |
| hAESCs | NCT04414813 | Stereotactic Transplantation of hAESCs for Parkinson's Disease | Parkinson’s disease – evaluation of stereotactic intraventricular transplantation of hAESCs | Early I | Completed | China |
| hAESCs | NCT03381807 | Human Amniotic Epithelial Stem Cell in Treatment of Refractory Severe Intrauterine Adhesion | Severe refractory Asherman’s syndrome – evaluation of intrauterine transplantation of hAESCs | Early I | Unknown status | China |
| hAESCs | NCT06444022 | hAESCs Prevent Acute Graft-versus-host Disease After Hematopoietic Stem Cell Transplantation | Acute GVHD prophylaxis following HSCT – evaluation of intravenous hAESC infusions | Early I | Not yet recruiting | China |
| hAMSCs – hFM-MSCs | NCT06891508 | The Placental Secretome as a Therapeutic Tool to Prevent Inflammation-induced Preterm Birth (PLACENTOMICS) | Preterm birth (PTB) associated with intra-amniotic inflammation (IAI) and/or preterm premature rupture of membranes (pPROM) | Not applicable | Active, not recruiting | Italy |
| hAMSCs | NCT06551649 | Human Amniotic Mesenchymal Cell Secretome for Neurodegeneration and Neuroinflammation (CONTRASTOME) | Neurodegenerative diseases (ALS and multiple sclerosis) – evaluation of hAMSC secretome in 3D in vitro models | Not applicable | Recruiting | Italy |
| hAMSCs | NCT04706312 | Transplantation of hAMSCs for Woman With DOR | Diminished ovarian response (DOR)-related infertility – evaluation of intravenous hAMSC infusion | I | Unknown status | China |
| hAMSCs | NCT07115082 | Clinical Study on the Safety and Efficacy of Human Amniotic Mesenchymal Stem Cells in the Treatment of Premature Ovarian Insufficiency | Primary ovarian insufficiency – evaluation of intravenous hAMSC infusion combined with hormone replacement therapy | I/II | Recruiting | China |
| Placental-Derived Mesenchymal Stem Cells (PMSCs) | NCT04453111 | Efficacy of Bone-marrow-derived and Placenta-derived Multipotent Mesenchymal Stem /​ Stromal Cells for Osteoarthritis | Knee osteoarthritis – evaluation of intra-articular transplantation of bone marrow- and placenta-derived MSCs | I/II | Unknown status | Ukraine |
| PMSCs | NCT06568653 | Human Placenta Mesenchymal Stem Cells Derived Exosomes Injection for Treatment of Complex Anal Fistula | Complex perianal fistula (non-Crohn’s) – evaluation of placenta-derived MSC exosome injections combined with fistulotomy | II | Not yet recruiting | Iran |
| AFSCs | NCT06386679 | Amniotic Fluid Mesenchymal Stem Cells Developed for Chondrogenic Treatment (AFCC) Injection in Elderly Knee Osteoarthritis Patients | Knee osteoarthritis – evaluation of intra-articular injection of amniotic fluid-derived mesenchymal stem cells (AF-MSCs) | I | Enrolling by invitation | Thailand |
| WJ-MSCs | NCT06812637 | Efficacy and Safety of Wharton's Jelly-Derived Mesenchymal Stem Cell Exosomes in the Treatment of Diabetic Foot Ulcers: A Double-blinded Randomized Controlled Clinical Trial (WJ-MSC) | Diabetic foot ulcers – evaluation of topical Wharton’s Jelly-derived MSC exosomes for wound healing | I | Completed | Egypt |
| WJ-MSCs | NCT03973827 | Wharton´s Jelly Derived Mesenchymal Stromal Cell Repeated Treatment of Adult Patients Diagnosed with Type I Diabetes | Type 1 diabetes mellitus – evaluation of repeated allogeneic WJ-MSC infusions to preserve endogenous insulin production | I/II | Completed | Sweden |
| WJ-MSCs | NCT06981338 | Guttmann NeuroRecovery - Viability, Safety, and Efficacy of Intrathecal Wharton's Jelly Mesenchymal Stem Cells and Transcutaneous Spinal Cord Stimulation in Chronic Spinal Cord Injury Rehabilitation (GNR-SCI-01) | Chronic traumatic spinal cord injury – evaluation of intrathecal Wharton’s Jelly-derived MSCs combined with transcutaneous spinal cord stimulation (tSCS) and neurorehabilitation | I/II | Not yet recruiting | Spain |
| WJ-MSCs | NCT05018637 | Efficacy of WJ-derived Mesenchymal Stem Cells in Combination With Parathyroid Hormone for Vertebral Compression Fracture | Osteoporotic vertebral compression fractures – evaluation of WJ-MSCs combined with teriparatide (PTH 1-34) | II | Unknown status | South Korea |
| WJ-MSCs | NCT03866330 | Wharton's Jelly-derived Mesenchymal Stem Cells in Osteoarthritis | Mild to moderate osteoarthritis of the hip, knee, or glenohumeral joint | I/II | Unknown status | Poland |
| WJ-MSCs | NCT03337243 | Effect of Implanting Allogenic Cytokines Derived From Human Amniotic Membrane (HAM) and Mesenchymal Stem Cells Derived From Human Umbilical Cord Wharton's Jelly (HUMCWJ) on Pain and Functioning of Knee Osteoarthritis | Knee osteoarthritis – evaluation of intra-articular implantation of allogenic cytokines derived from human amniotic membranes (HAM) and mesenchymal stem cells derived from Wharton’s Jelly (WJ-MSCs) | Not applicable | Completed | USA |
| WJ-MSCs | NCT03158896 | Evaluation of Umbilical Cord-Derived Wharton's Jelly Stem Cells for the Treatment of Acute Graft Versus Host Disease | Acute graft-versus-host disease (aGVHD) after allogeneic hematopoietic stem cell transplantation | I | Recruiting | USA |
| WJ-MSCs | NCT05855707 | Wharton Jelly Mesenchymal Stromal Cells as GVHD Prophylaxis (HAPLO-GEL) | Advanced hematological malignancies undergoing haploidentical allo-SCT – GvHD prophylaxis and hematopoietic support | I | Not yet recruiting | France |
| WJ-MSCs | NCT04625738 | Efficacy of Infusions of MSC From Wharton Jelly in the SARS-Cov-2 (COVID-19) Related Acute Respiratory Distress Syndrome (MSC-COVID19) | COVID-19-related moderate to severe ARDS requiring mechanical ventilation | II | Completed | France |
| WJ-MSCs | NCT02368587 | Intracoronary or Intravenous Infusion Human Wharton' Jelly-derived Mesenchymal Stem Cells in Patients With Ischemic Cardiomyopathy (WJ-ICMP Tria) | Ischemic heart failure after myocardial infarction – evaluation of intracoronary or intravenous Wharton’s Jelly-derived MSC infusion | II | Unknown status | China |
| WJ-MSCs | NCT04551456 | WJMSCs Anti-inflammatory Therapy in Coronary Artery Disease (WANICHD) | Coronary artery atherosclerosis – evaluation of intravenous Wharton’s Jelly-derived MSC infusions for anti-inflammatory therapy | II | Unknown status | China |
| WJ-MSCs | NCT06146062 | Effects of Intravascular Administration of Mesenchymal Stromal Cells Derived from Wharton's Jelly of the Umbilical Cord on Systemic Immunomodulation and Neuroinflammation After Traumatic Brain Injury. (TRAUMACELL) | Severe traumatic brain injury – evaluation of iterative intravenous Wharton’s Jelly-derived MSC infusions for post-traumatic neuroinflammation and neurological recovery | II | Recruiting | France |
| WJ-MSCs | NCT04651855 | The Evaluation of the Effect of Mesenchymal Stem Cells on the Immune System of Patients With ALS (ALSTEM) | Amyotrophic lateral sclerosis (ALS) – evaluation of intrathecal Wharton’s Jelly-derived MSC administration | I/II | Unknown status | Poland |
| MenSCs | NCT05019287 | Menstrual Blood Stem Cells in Severe Covid-19 | Severe COVID-19 – evaluation of menstrual blood stem cell-derived secretome for cytokine storm modulation and lung regeneration | I/II | Completed | Iran |
| MenSCs | NCT05703308 | Menstrual Blood Stem Cells in Poor Ovarian Responders | Poor ovarian response (POR) in infertile women – evaluation of intraovarian autologous menstrual blood-derived stem cell transplantation | III | Completed | Iran |
| MenSCs | NCT07131150 | Menstrual Blood-Derived Mesenchymal Stem Cell Injection (SC01009) in the Treatment of Idiopathic Pulmonary Fibrosis (IPF II) | Idiopathic Pulmonary Fibrosis (IPF) – evaluation of SC01009 (menstrual blood-derived MSCs) for improving lung function and disease progression | II | Not yet recruiting | China |
| DPSCs | NCT05924373 | Human Dental Pulp Mesenchymal Stem Cells for the Treatment of Chronic Periodontitis Patients | Chronic periodontitis – evaluation of safety and efficacy of different administration protocols of human dental pulp mesenchymal stem cells (hDP-MSCs) through local injection | II | Recruiting | China |
| DPSCs | NCT04983225 | The Safety and Tolerability of Initial Periodontal Therapy Combined With Human Dental Pulp Stem Cell Injection in the Treatment of Chronic Periodontitis | Chronic periodontitis – evaluation of safety, tolerability, and preliminary efficacy of human dental pulp mesenchymal stem cell (hDP-MSC) injections as adjunct to initial periodontal therapy | I | Unknown status | China |
| DPSCs | NCT04130100 | Clinical Study of Pulp Mesenchymal Stem Cells in the Treatment of Primary Mild to Moderate Knee Osteoarthritis | Mild to moderate primary knee osteoarthritis – evaluation of safety and clinical efficacy of dental pulp mesenchymal stem cells (hDP-MSCs) compared with sodium hyaluronate | Early I | Unknown status | China |
| DPSCs | NCT03912480 | Stem Cells From Human Exfoliated Teeth in Treatment of Diabetic Patients With Significantly Reduced Islet Function | Diabetes mellitus with significantly reduced islet function – evaluation of safety and efficacy of stem cells from human exfoliated teeth (SHED) transplantation as adjunct to standard therapy | Early I | Unknown status | China |
| DPSCs | NCT04336254 | Safety and Efficacy Study of Allogeneic Human Dental Pulp Mesenchymal Stem Cells to Treat Severe COVID-19 Patients | Severe COVID-19 pneumonia – evaluation of safety and efficacy of allogeneic human dental pulp mesenchymal stem cells (hDP-MSCs) for reducing mortality and improving clinical prognosis | I/II | Unknown status | China |
| DPSCs | NCT04608838 | A Randomized Placebo-controlled Multicenter Trial to Evaluate the Efficacy and Safety of JTR-161, Allogeneic Human Dental Pulp Stem Cell, in Patients With Acute Ischemic stRoke (J-REPAIR) (J-REPAIR) | Acute ischemic stroke – evaluation of safety and efficacy of a single intravenous administration of JTR-161 (allogeneic dental pulp-derived mesenchymal stem cell product) | I/II | Completed | Japan |
